# Putative Antimicrobial Peptides Within Bacterial Proteomes Affect Bacterial Predominance: A Network Analysis Perspective

**DOI:** 10.3389/fmicb.2021.752674

**Published:** 2021-11-12

**Authors:** Anastasis Oulas, Margarita Zachariou, Christos T. Chasapis, Marios Tomazou, Umer Z. Ijaz, Georges Pierre Schmartz, George M. Spyrou, Alexios Vlamis-Gardikas

**Affiliations:** ^1^Bioinformatics Department, The Cyprus Institute of Neurology and Genetics, Nicosia, Cyprus; ^2^The Cyprus School of Molecular Medicine, Nicosia, Cyprus; ^3^NMR Center, Instrumental Analysis Laboratory, School of Natural Sciences, University of Patras, Patras, Greece; ^4^School of Engineering, University of Glasgow, Glasgow, United Kingdom; ^5^Chair for Clinical Bioinformatics, Saarland University, Saarbrücken, Germany; ^6^Division of Organic Chemistry, Biochemistry and Natural Products, Department of Chemistry, University of Patras, Patras, Greece

**Keywords:** putative antimicrobial peptides, interbacterial antagonism, network analysis, bioinformatics analysis, bacterial competition

## Abstract

The predominance of bacterial taxa in the gut, was examined in view of the putative antimicrobial peptide sequences (AMPs) within their proteomes. The working assumption was that compatible bacteria would share homology and thus immunity to their putative AMPs, while competing taxa would have dissimilarities in their proteome-hidden AMPs. A network–based method (“Bacterial Wars”) was developed to handle sequence similarities of predicted AMPs among *UniProt*-derived protein sequences from different bacterial taxa, while a resulting parameter (“*Die*” score) suggested which taxa would prevail in a defined microbiome. T he working hypothesis was examined by correlating the calculated *Die* scores, to the abundance of bacterial taxa from gut microbiomes from different states of health and disease. Eleven publicly available 16S rRNA datasets and a dataset from a full shotgun metagenomics served for the analysis. The overall conclusion was that AMPs encrypted within bacterial proteomes affected the predominance of bacterial taxa in chemospheres.

## Introduction

In terms of species and population, bacterial communities occur in dynamic equilibria related to environmental factors that drive interspecies competition and coexistence. Different species may facilitate their survival in a particular environment (e.g., gut and biofilms) by sharing information on existing sources of food or threats using small molecular messengers (Mukherjee and Bassler, 2019). Apart from aiding each other, bacteria most often compete using toxic molecules. In a close contact intercellular war, they may inject lethal molecules (peptidoglycan hydrolases, phospholipases, pore forming proteins, DNases, RNases, NAD(P)^+^ hydrolases, and ADP-ribosyltransferase among others), to suppress antagonistic species using the type VI secretion system (Coulthurst, 2019; Ross et al., 2019). Another type of antagonism is the secretion of toxic molecules destined for competitors (Richards et al., 2017). If the toxic effector molecules are proteinaceous they are called bacteriocins (Cotter et al., 2013). The term describes a broad and heterogenous category of molecules that can inhibit bacterial growth while being present in a defined contained environment (chemosphere) (Baquero et al., 2019). They are roughly divided in two categories: one of transmembrane proteins [colicins (Kleanthous, 2010)] and another of peptides (class I bacteriocins) (Riley and Wertz, 2002). Microcins are class I bacteriocins with pluripotent inhibitory actions that may act in synergy with the larger colicins for even broader inhibition effects (Baquero et al., 2019). Bacteriocins have a narrow target range and are designed to benefit the species that produces them over its closely related competitors.

Antimicrobial peptides (AMPs), a term first used for antibacterial peptides of the innate immune response of eukaryotes, are found in all kingdoms of life (Hancock, 2001). Eukaryotic AMPs mostly target and disrupt bacterial membranes at micromolar concentrations, without interacting with receptors (Kumar et al., 2018). They take advantage of the specific lipid constitution of bacterial membranes [very polar parts of the exposed lipid heads (Kumar et al., 2018)] and the curvature of the bacterial membrane (Matsuzaki et al., 1998; Drin and Antonny, 2010) to act as detergents lysing the bacterial cell. AMP-like detergents may constitute part of bacterial defenses against other bacterial species [e.g., class I bacteriocin nisin from *Lactococcus lactis* (Breukink et al., 1999), mersacidin from *Bacillus* sp. (Chatterjee et al., 1992), bacteriocins MccV, MccE492, and MccL]. The bacteria generating AMPs are not harmed due to specific immunity mechanisms that include specific AMP pumps (Beis and Rebuffat, 2019), or dedicated immunity proteins embedded within the cellular membrane, that specifically bind to the cognate AMPs on the extracellular side preventing its entrance-damage to the cell (Hassan et al., 2012; Beis and Rebuffat, 2019).

Antimicrobial peptides maintain a balanced microbiome and establish compatibility between bacterial populations in niches such as the human gut (Kerr et al., 2002; Kirkup and Riley, 2004; Majeed et al., 2011). Apart from their role in microbial biodiversity, AMPs have been employed as antimicrobials. Purified AMPs have been used to extend food preservation time, in the treatment of infectious diseases (e.g., skin infections and wounds) (Pfalzgraff et al., 2018) and cancer (Rodrigues et al., 2019). The therapeutic potential of AMPs as a replacement drug candidate for antibiotics has immense potential, in particular for the treatment of pathogens resistant to antibiotics (Yang et al., 2014).

Antimicrobial peptides that form pores are generally considered α-helical amphipathic molecules that may act as detergents on bacterial membranes (Blondelle et al., 1999). The amphipathicity and α-helical secondary structure characteristics have been exploited by a number of prediction tools in order to predict AMPs in a given protein sequence (Gabere and Noble, 2017). Detailed studies on mutations affecting the toxicity of eukaryotic bovine peptide bactenecin 2A (Hilpert et al., 2005) have been used for the formulation of *AMPA*, a predictor of antimicrobial peptides in a protein sequence (Torrent et al., 2009, 2012). Specifically, *AMPA* uses a sophisticated algorithm that gives an antimicrobial propensity value for a selected peptide stretch (Torrent et al., 2009). Each amino acid of the stretch is assigned an “antimicrobial index” value derived from high-throughput screening results (antimicrobial IC_50_ values) concerning amino acid replacements of the AMP bactenecin 2A (Hilpert et al., 2005). *AMPA* has been thoroughly validated *in silico* exhibiting accurate prediction of 80–90% of the assessed antimicrobial proteins including human ribonucleases, lysozymes and bacterial bacteriocins (Torrent et al., 2009).

We wondered whether bacterial proteomes might hide antibacterial stretches that could be used for interbacterial competition among different genera. Assuming that sequence similarities of AMPs from the proteomes of different bacteria could form a basis for interspecies competition, a computational method termed Bacterial Wars (BW) was developed and employed herein to explore the relationship between putative AMPs and bacterial predominance. The BW method (i) analyzed and compared putative AMPs hidden in the proteomes of different bacterial taxa and (ii) used these AMPs to predict interbacterial antagonism based on their sequence similarities. Finally, we put the hypothesis to the test by comparing the outcome of BW, to the abundance of bacterial genera in eleven publicly available 16S rRNA derived datasets as well as the species from a high-resolution full shotgun metagenomics dataset. The overall outcome was that AMPs embedded within bacterial proteomes may affect prevalence of individual taxa in the gut. To differentiate this form of competition from previously known specialized mechanisms, the novel concept is coined as “putative AMP defense.”

## Results

### Large Scale Prediction of Bacterial Antimicrobial Peptides, and Construction of Bacterial Networks – The Bacterial Wars Method

**FIGURE 1:**
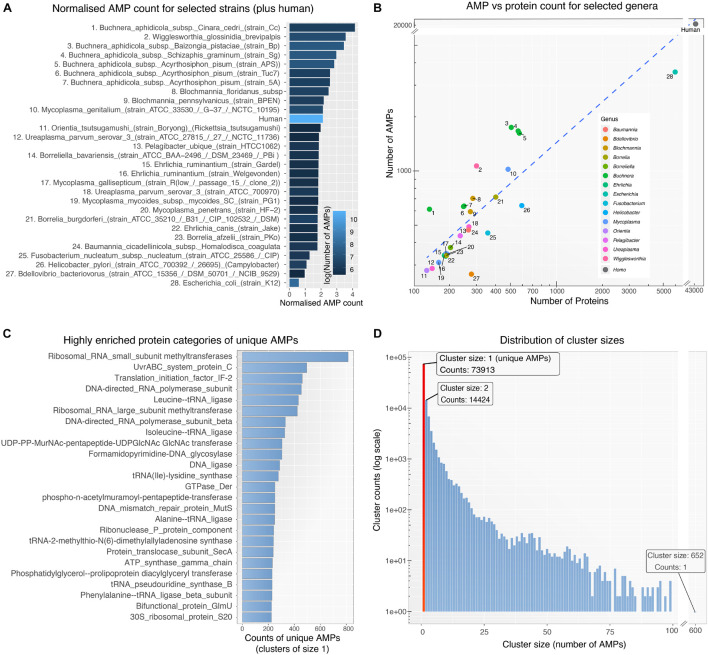
**(A)** Normalized AMP counts for bacterial species with the highest number of predicted AMPs (1–25), selected species of interest (26–28) and human. AMP counts were normalized by the number of proteins included in the prediction process (AMP/protein ratio). Due to filtering of proteins (only curated *Swiss-Prot* taken in consideration) some species had very few remaining proteins. For visualization purposes, only species with at least 140 remaining proteins are shown above. The color scale represents the actual number of AMPs for each organism. **(B)** Scatter plot showing the number of predicted AMPs against the number of proteins used as input to *AMPA* software for each organism. Numerical labels correspond to the species in 1a while color groups represent taxa. **(C)** The 25 most enriched protein categories derived from clusters of size 1 (i.e., unique AMPs). **(D)** Histogram for all 109.678 clusters showing the distribution of clusters size (i.e., number of AMPs within the cluster – AMPs with >80% sequence similarity). The red bar highlights the large number of unique AMPs (cluster size of 1).

**FIGURE 2:**
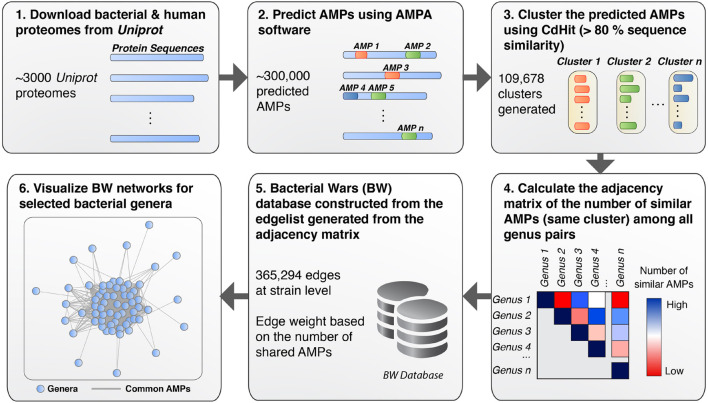
Schematic workflow of the BW methodology. (1) All bacterial proteomes were retrieved from *UniProt* and filtered to extract the *Swiss-Prot* curated sequences. (2) AMP perdition was performed using *AMPA*. (3) *CdHit* was used to cluster AMPs based on sequence similarity (threshold >80%). (4) Pairwise comparison was performed for all bacterial taxa to obtain the number of common AMPs for each bacterial pair (i.e., creating an adjacency matrix). (5) The adjacency matrix was used to construct an edge list, which, was used to create the BW database of interactions. (6) Querying the BW database for selected taxa allows for networks of interactions to be generated where bacteria taxa are represented as nodes and the number of common peptides as the edge weight.

To detect and analyze putative AMPs from different bacterial species we first created a database (available as a data list – see below for details) that related bacterial species by the sequence similarities of their AMP. The database was then queried to form networks of bacterial interactions based on the number of common AMPs (details below). To achieve these goals and develop the BW method, the following steps were performed: (1) All proteomes from ∼3000 bacterial strains were downloaded from *UniProt*. Only *Swiss-Prot* sequences were retained from the proteomes as these are fully curated. (2) The curated protein sets of these species were used as input for the *AMPA* software (Torrent et al., 2009, 2012), which predicted and assigned a propensity score to all putative AMP sequences. Over 300,000 AMPs were predicted for all bacterial species, with some showing higher numbers of predicted AMPs with respect to others (Figure 1A). Although bacterial putative AMPs were in high numbers they trailed behind the number of putative AMPs from human (Figures 1A,B). Still their numbers were significantly higher than the 200 experimentally verified peptides attributed so far to all bacterial species (Kumar et al., 2018). Certain species (e.g., *Buchnera aphidicola*) deviated from the fitted dashed regression line, highlighting a disproportional ratio of predicted AMPs (high) with respect to proteins included in the analysis. (3) The resulting bacterial AMPs were next fed into *CdHit* (Li and Godzik, 2006; Fu et al., 2012) to obtain clusters of highly similar peptides (>80% sequence similarity). This resulted in 109,678 clusters, most of which were comprised of solely one member, namely AMPs which are unique to one species. Further analysis of the unique AMPs revealed that they were encrypted in specific protein categories, especially proteins binding to nucleic acids (Figure 1C). A full list of all the protein description in clusters with unique AMPs are available as Supplementary Table 1. Histograms showing the overall cluster distribution obtained from *CdHit* also highlight a large portion of unique AMPs (Figure 1D). (4) Next, an adjacency matrix was created by calculating the number of highly similar/common AMPs between all pairs of species in our dataset. (5) This matrix was later used to create an edge list for the construction of the BW database (the edge list is available as Supplementary Table 2). (6) BW utilized this information to construct and visualize genus-to-genus (which can also extend to species-to-species) networks. Network nodes denote species or taxa and edges denote nodes with common AMPs. The weight of the edges is proportional to the number of shared common AMPs. The overall methodology is described schematically in Figure 2.

### A Network Measure to Assess Bacterial Antagonism – The *Die* Score

Following the generation of networks of bacterial relationships based on their common AMPs, a novel network-based score was introduced (termed *Die* score). The *Die* score was used for calculating the relative likelihood for any given bacterial species (node) to die in a specific microbiome. We hypothesized that since bacteria are immune to their own AMPs, taxa that shared many common AMPs (high edge weight) would compete less. Therefore, the capacity of bacterial taxa to antagonize each other should be inversely dependent on the similarity of their AMPs. The *Die* score can quantify this hypothesis using network topology calculations, thus providing measure of how likely a bacterial node will die in the existing network of bacterial interactions. The higher (more positive) the *Die* score, the more likely for a species to die in the underlining microbiome, while smaller (or negative) *Die* scores exhibit the opposite trend. A mathematical explanation of the *Die* score and how it is calculated for all nodes in a given network is described in the section “Materials and Methods.”

### Validation Schemes of the Putative Antimicrobial Peptide Defense Hypothesis

#### Validation 1 – Building of Bacterial Wars and Microbiome Networks From Publicly Available Data

**FIGURE 3:**
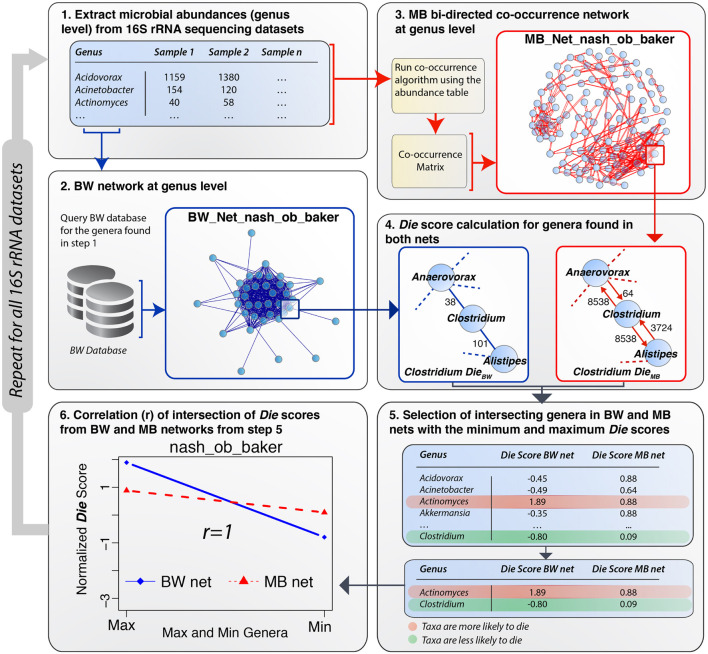
Schematic workflow of the BW validation process. (1) A list of microbial genera was extracted from microbiome dataset (e.g., Zhu et al. (2013) NASH vs. Control gut MB dataset). (2) The BW database was queried using this list and the relevant BW network was constructed from the results obtained from this query. (3) A MB co-occurrence bi-directed network using a *rho = 0.4* and *p*-value = *0.05* was constructed from the abundance table for the case study dataset. (4) *Die* scores were calculated for both networks and normalized by subtracting the mean and dividing by the standard deviation. (5) Some bacterial genera were not included in the BW database and therefore only the intersection of *Die* scores (full list in Supplementary Table 3) from both networks was taken into consideration in step 5. The rows in the table are highlighted in red (maximum, positive *Die* score) and green (minimum, negative *Die* score), following the legend shown in the bottom right panel. The transition from red to green denotes genera, which are more or less likely to die, respectively. (6) Pearson’s correlation coefficient was calculated for the BW min/max *Die* scores obtaining an *r-*value of 1.

Having constructed a methodology (BW) to extract and analyze relationships among different proteomes according to their predicted AMPs, validation of the outcome was performed using MB data from eleven 16S rRNA gene plus 1 shotgun gut microbiome datasets (Duvallet et al., 2017). The MB data provide an accurate representation of actual bacterial abundances in the underlying microbiomes. All bacterial abundances were extracted at the genus level from each 16S rRNA gene MB dataset and at the species level for the shotgun MB dataset. Two parallel approaches were performed using the *Die* score, one for BW and one for MB–generated networks. A detailed overview of the validation process using one of the microbiomes [Zhu et al. (2013), non-alcoholic steatohepatitis (NASH) vs. Healthy] (Zhu et al., 2013) was performed as a case study (Figure 3). The process involved six steps: (1) a list of bacterial genera was extracted from the Zhu et al. (2013), NASH dataset and used to query the BW database (edge list file) to obtain information on the number of shared common peptides between all pairs of bacterial genera in the microbiome list. (2) A network of bacteria genera, termed the BW network, was constructed using the information available from the BW database. Genera were denoted as network nodes and the number of common AMPs defined their edge weights. Similarly, (3) a network of co-occurrence was created using the abundance data available for the Zhu et al. (2013), NASH dataset, termed the MB network. Co-occurrence networks were constrained by different correlation strengths (*rho*) including both positive co-occurrence (+*rho*) and negative co-occurrence (−*rho*) networks. Different *rho* cut-offs were set according to the size of the dataset in-hand. The co-occurrence network was used as the validation network. (4) For an examined dataset, *Die* scores were calculated for all the nodes in both the BW network and the MB network. The MB network did not provide any information on AMPs. To render it comparable with the BW network, we transformed the MB network to a bi-directed network where the outgoing edge weights represented the abundance of the bacterial genus at any given node (see subnetworks in Figure 3, step 4). In the bi-directed MB network, high weight of an outgoing edge (high abundance), denoted greater compatibility within a particular microbiome. Inversely, low weights of an outgoing edge (lower abundance), denoted less compatibility among bacterial genera within the microbiome under investigation. The construction of the bi-directed MB network allowed for the application of the *Die* score to both BW and MB networks, resulting in two lists of *Die* scores. (5) An intersection of the two types of networks was obtained, as some genera were not included in the BW database. A partial table with the lists obtained from the intersection of *Die* scores is shown in step 5 of Figure 3. Supplementary Table 3 shows the full lists of genera and their *Die* scores, for both the BW and BW networks for the case study by Zhu et al. (2013), as well as all additional datasets analyzed herein. Finally, (6) the correlation between the *Die* score lists from both networks was calculated to provide a statistical significance for the validity of the working hypothesis (see next section for detailed results).

#### Validation 2 – Comparison of *Die* Scores Between Bacterial Wars and Microbiome Networks by the Pearson’s Correlation Coefficient

The two *Die* score lists generated from the taxa of the BW and MB networks (Figure 3 steps 5–6) were compared using the Pearson’s correlation coefficient. A positive correlation would provide evidence in favor of the putative AMP defense hypothesis. Initially, taxa with the highest (maximum) and lowest (minimum) *Die* scores from a given BW network were compared to the *Die* scores from the respective MB network.

A complete agreement was observed in the correlation coefficient (*r* = 1) across all eleven 16S microbiome datasets using this initial assessment (Supplementary Figure 1). However, correlation was not observed for the shotgun metagenomics dataset at the species level. When taxa with the second highest and lowest *Die* scores were compared, the correlation dropped for some datasets, averaging a correlation coefficient of *r* = 0.425 across all twelve microbiomes. Similarly, when the comparison was extended to include taxa with the third highest and lowest *Die* scores, Pearson’s correlations averaged a total of *r* = 0.325 across all datasets (Supplementary Figure 1). Thus, BW and MB data were in agreement for genera with the highest (maximum) and lowest (minimum) *Die* scores but not for all taxa with intermediate *Die* score values and not for individual species.

#### Validation 3 – Assessing the Skewness of *Die* Score Distributions for Bacterial Wars and Microbiome Networks

**FIGURE 4:**
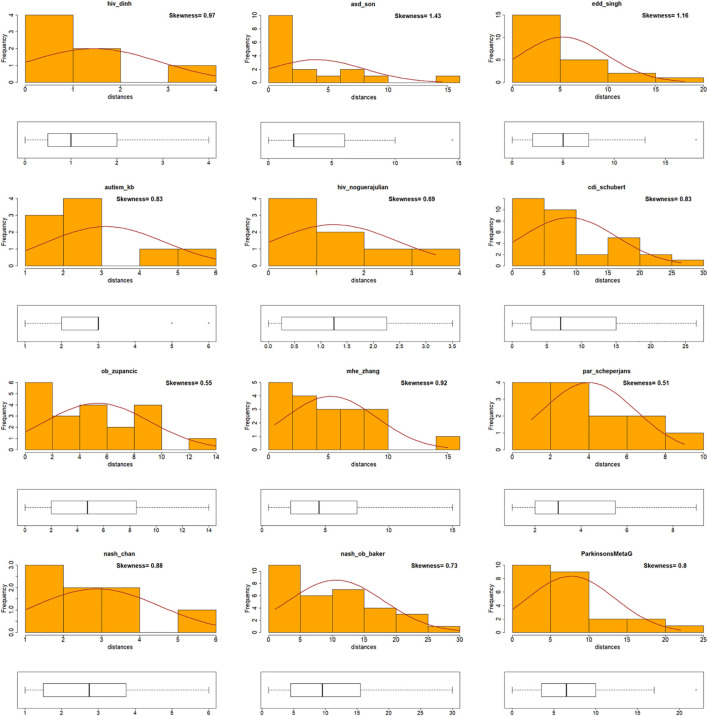
Visualization of skewness for *Die* score differences for BW and MB networks. Histogram plots for all the data (disease and control) in twelve microbiomes [including Zhu et al. (2013) dataset – labeled as nash_ob_baker], showing the distribution and skewness of the distances (*Dist*). Positively skewed distributions of *Dist* measures were observed for all twelve microbiomes.

A correlation coefficient provides an adequate insight of the trend of the data. However, if a specific genus or species achieves a high *Die* score in both BW and MB networks but does not appear in the same ranking in both networks, then it would not adhere to the correlation trend. This would result in a poor Pearson’s correlation coefficient, even though the underlined genus/species scores were high in both incidents. To obtain insights that a Pearson’s correlation coefficient measure could not provide, the shape of distributions of the *Die* score values (skewness) was examined. First, taxa were ranked according to their *Die* scores as derived from the two networks and the absolute distance of the rankings was calculated (see Table 1). The new measure (*Dist*) provided an indication of the difference in magnitude between the *Die* scores from the two different networks. Data that were positively skewed with respect to their distances (*Dist*) indicated that there was a small difference between the taxa rankings based on their *Die score.* For example, taxa with high or low *Die* scores in one distribution (i.e., BW), also achieved proportionally high or low *Die* scores in the second distribution (i.e., MB). This would provide evidence to support the validity of the putative AMP defense hypothesis and would agree with bacterial predominance based on proteome-derived AMPs as a potential mechanism for bacterial prevalence in a specific microbiome. On the other hand, data with a negatively skewed *Dist* would mean that the *Die* score rankings had large differences. For example, taxa with high or low *Die* scores under one distribution, would achieve inversely proportional low and high scores in the other. This would provide evidence against the validity of the putative AMP defense hypothesis. Table 1 shows the procedure of calculating the absolute distance (*Dist*). A full list of all the taxa can be found as Supplementary Table 3. The use of the *Dist* measure to investigate skewness for the twelve microbiome datasets showed that they adhered to positively skewed distributions with respect to their *Dist* measure (Figure 4). Therefore, skewness comparisons showed a notable agreement for the BW and MB networks that previously seemed not to correlate using the Pearson’s correlation coefficient only.

**TABLE 1:** Absolute distance (*Dist*) calculation using Zhu et al. (2013) NASH dataset as a case study.

	Die_BW_ score	Die_MB_ score	Die_BW_ ranks	Die_MB_ ranks	Dist
*Acidovorax*	−0.45	0.88	20	32	12
*Acinetobacter*	−0.49	0.64	14	21	7
*Actinomyces*	1.89	0.88	32	28	4
*Akkermansia*	−0.35	0.88	26	25	1
*Arcobacter*	−0.43	−0.58	24	7	17
*Bifidobacterium*	−0.54	−0.01	11	10	1
*Campylobacter*	−0.46	0.86	18	23	5
*Citrobacter*	−0.56	0.88	10	31	21
*Clostridium*	−0.8	0.09	1	12	11
*…*	…	…	…	…	…
*Weissella*	−0.35	0.4	25	18	7

#### Validation 4 – Statistical Analysis of the *Die* Scores Between Bacterial Wars and Microbiome Networks by the Wilcoxon Test

**FIGURE 5:**
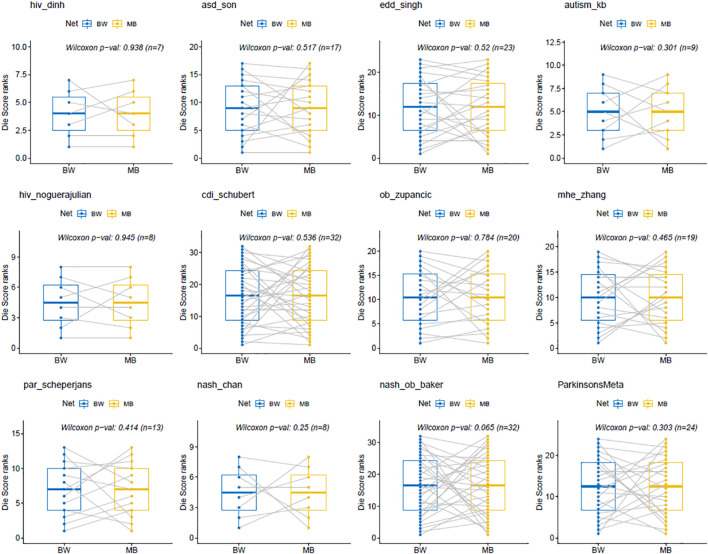
Wilcoxon signed-rank test for BW and MB networks. The *p*-values are all >0.05, indicating that the results obtained from the Wilcoxon tests using the ranked *Die* scores were not statistically significant between the BW and MB networks. This is in favor of the putative AMP defense hypothesis that would expect similar distributions for both types of *Die* score lists.

Statistical analysis of the *Die* scores between BW and MB networks was performed by the Wilcoxon signed-rank test. This is a non-parametric statistical test for paired groups and was used to compare the two *Die* score lists and assessed whether the ranking order of their values differed. The differences in the rankings of the *Die* scores from the BW and MB networks across all twelve MB datasets (Figure 5), were not statistically significant (*p*-values > 0.05). In other words, there was no evidence to reject the null hypothesis stating that the two *Die* score lists have the same continuous distribution. This provides evidence in favor of the putative AMP defense hypothesis, according to which the *Die* score list distributions between BW and MB networks should be similar.

#### Validation 5 – Comparisons of *Die* Scores Between Bacterial Wars and Microbiome Networks From States of Health and Disease

*Die* scores from BW networks and MB co-occurrence networks for twelve gut microbiome datasets were positively correlated (positive skewness for *Dist*). In these comparisons, all healthy and all diseased state microbiomes for each of the 12 datasets were grouped together (the analyzed MB datasets contained both disease and control samples). Next, we examined whether correlations would be affected upon separation of the MB data into different sample types with greater homogeneity. Therefore, the analysis steps outlined in Figure 3 were repeated after separating the twelve gut MB data to their health and diseased states.

##### Case Study 1 – Healthy States

Taxa with the highest (maximum) and lowest (minimum) *Die* scores from the BW network were positively correlated to the *Die* scores from the corresponding taxa from the MB network for 10 out of 12 datasets (Table 2A). In addition, the skewness of the different *Die* scores was also positively skewed for 12 out of 12 (100%) of the MB-control networks when compared to the corresponding BW network’s *Die* scores (Supplementary Figure 2 and Supplementary Table 4).

**TABLE 2:** Top *Die* score taxa for healthy and disease MB networks and their correlation with BW *Die* scores.

**(A) Healthy states**			

Dataset	Most likely to die	Least likely to die	Pearson’s correlation *Die*_BW_ vs. *Die*_MB_
HIV_Dinh	*Pseudobutyrivibrio*	*Clostridium*	1
ASD_Son	*Prevotella*	*Salmonella*	−1
EDD_Singh	*Actinomyces*	*Pseudomonas*	1
ASD_KB	*Eubacterium*	*Bacteroides*	1
HIV_Noguera-Julian	*Tannerella*	*Streptococcus*	1
CDI_Schubert	*Actinomyces, Anaerobiospirillum*	*Neisseria*	−0.8
OB_Zupancic	*Dickeya*	*Pseudomonas*	1
MHE_Zhang	*Dickeya*	*Enterobacter*	1
PAR_Scheperjans	*Hafnia*	*Salmonella*	1
NASH_Chan	*Prevotella*	*Haemophilus*	1
NASH_OB_Baker	*Prevotella*	*Streptococcus*	1
ParkinsonsMetaG	*Lactobacillus acidophilus*	*Staphylococcus aureus*	1

**(B) Diseased states**			

**Dataset**	**Most likely to die**	**Least likely to die**	**Pearson’s correlation *Die*_BW_ vs. *Die*_MB_**

HIV_Dinh	*Pseudobutyrivibrio*	*Clostridium*	1
ASD_Son	*Comamonas*	*Streptococcus*	1
EDD_Singh	*Anaeroplasma, Tannerella*	*Yersinia*	1
ASD_KB	*Prevotella*	*Lactobacillus*	1
HIV_Noguera-Julian	*Anaerobiospirillum, Ensifer*	*Streptococcus*	1
CDI_Schubert	*Carnobacterium*	*Pseudomonas*	1
OB_Zupancic	*Pseudobutyrivibrio*	*Haemophilus*	1
MHE_Zhang	*Leptotrichia*	*Streptococcus*	1
PAR_Scheperjans	*Pseudobutyrivibrio*	*Streptococcus*	1
NASH_Chan	*Salmonella*	*Bifidobacterium*	−1
NASH_OB_Baker	*Actinomyces*	*Streptococcus*	1
ParkinsonsMetaG	*Alcaligenes faecalis*	*Staphylococcus aureus*	−1

##### Case Study 2 – Diseased States

Performing the same analysis on the diseased state microbiome datasets, taxa with the highest (maximum) and lowest (minimum) *Die* scores from the BW network were in overall positive correlation (10 out of 12 datasets) with the *Die* scores from the corresponding taxa from the MB network. An exception was the microbiome data set from the nash_chan dataset (Table 2B). In addition, the different *Die* scores were also positively skewed for 9 out of 12 (75%) of the MB-diseased state networks (Supplementary Figure 3 and Supplementary Table 4).

In both case studies, taxa with high and low *Die* scores were different in the healthy and diseased state networks. This highlights the potential of the BW method to follow-up/predict taxa, which are more, or less likely to die in a given gut microbiome. All correlation plots for the datasets analyzed herein can be found as Supplementary Figures 4–6. Diseased state bacterial taxa corroborated by bibliographic information can be viewed in Supplementary Table 5.

### Evolutionary Considerations

**FIGURE 6:**
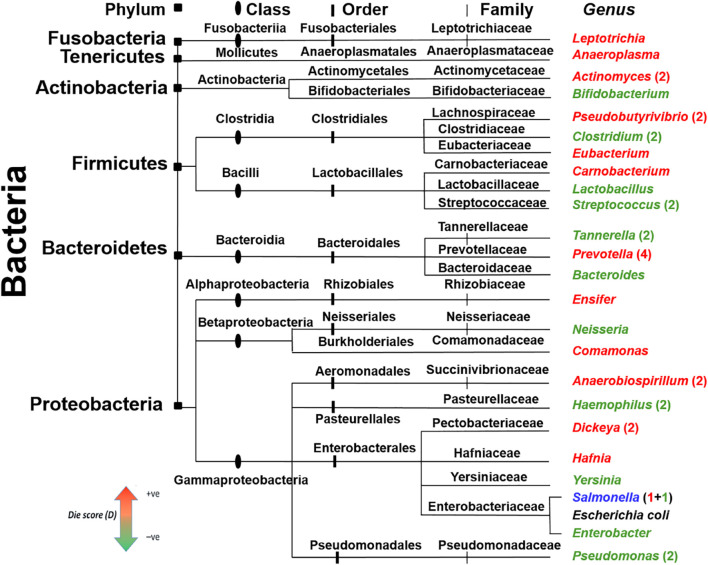
Phylogenetic distribution of the bacteria with the maximum (red) and minimum (green) *Die* scores. Bacterial genera were taken from controls and disease data, Tables 2A,B and were colored according to their BW *Die* scores. The taxonomic division for the different genera is shown on top and depicted as notches of different shapes on the ancestry lines. *Escherichia coli* (black color text) is included for comparison. Genera without numbers represent single observations from Tables 2A,B. When present, numbers represent more observations. *Salmonella* (blue) acquired low and high *Die* scores in two different microbiomes.

In the BW methodology, clustering of highly similar peptides was based on >80% sequence similarity. This high threshold raised the possibility of a similar clustering of AMPs from genera with close phylogenetic origin. To address this issue, a phylogenetic tree of all genera used in the case study for healthy and diseased state microbiomes (Tables 2A,B) was constructed along with the *Die* score as predicted by BW (Figure 6). According to the presented distribution, bacteria of the same family did not have similar *Die* scores. This observation demonstrates that the putative AMP defense hypothesis is not biased by bacterial genera with high evolutionary conservation and therefore high genomic sequence similarity. In fact, genera such as *Prevotella*, *Tannarella*, and *Bacteroides*, fared differently within the specific microbiomes presented here, despite all being members of the order Bacteroidales. *Prevotella* obtained high *Die* score (more likely to die) while the latter two attained low *Die* scores (less likely to die). Different *Die* scores were obtained for *Pseudobutyrivibrio*, *Clostridium*, and *Eubacterium* from Clostridiales and *Carnobacterium*, *Lactobacillus*, and *Streptococcus* of Lactobacillales. These findings demonstrate the extreme specificity of different AMPs for different genera.

## Discussion

The concept of peptides of bacterial origin as factors of interbacterial antagonism is not new (Baquero and Moreno, 1984). Examples date back to microcin E492 which is secreted by *Klebsiella pneumoniae* RYC492 and is active against various *Enterobacteriaceae* (de Lorenzo, 1984; Thomas et al., 2004; Gillor et al., 2008). Some of these peptides [e.g., mersacidin, nisin, subtilin, cinnamycin, duramycin, actagardine, epidermin, gallidermin, lanthiopeptin (Chatterjee et al., 1992)], may require post translational modification [addition of lanthionine (Knerr and van der Donk, 2012)] to stabilize the peptide and enhance the killing effect. Synthetic improvements of hairpin AMPs have also been used (Wu and Hancock, 1999; Liu et al., 2013). Despite the wide diversity of bacterial species, the isolated and characterized AMPs from bacteria are a fraction compared to those from eukaryotes (200 bacterial AMPs compared to 2159 from animals) (Kumar et al., 2018). The herein predicted 3000 putative AMPs for *E. coli* only (Figure 1B), suggests that the number of bacterial (and human) AMPs may be underestimated. The high number of bacterial AMPs is suggestive of a possible underlying function. Could it be that bacteria (and perhaps other organisms) employ AMPs encrypted in their proteomes to defend themselves against foe, especially in the relatively stable environment of a chemosphere (e.g., gut)? Could it be that a dying or dead bacterium might become a source of AMPs resulting from the proteolysis of its own proteins, to antagonize “enemy” species? This possibility would require the presence of proteases in a chemosphere. Bacterial proteases do exist in the colon (Ruiz-Perez and Nataro, 2014; Sears et al., 2014; Guyton et al., 2019) as well as proteases from other microorganisms (Espinosa-Cantellano and Martínez-Palomo, 2000; Ajjampur and Tan, 2016) to account for such proteolytic activity. Most of the proteins identified as potential sources of AMPs were cationic with nucleic acid binding properties (Figure 1B). Such proteins contain many basic residues (e.g., lysines) and are thus amenable to proteolytic degradation. The concept of AMP homology among bacterial proteomes as being the main prerequisite for bacterial species compatibility cannot be envisaged without proteolysis occurring in a chemosphere that would allow for the continuous production of AMPs. A mechanism as such would relieve the cell from the need of the energy expenses to synthesize *de novo* AMPs by protein synthesis.

Under the supposition that a bacterial proteome is a potential source of AMPs that are not toxic to the host species and/or species that share AMP homology, a methodology was developed (“Bacterial Wars” or BW) that could predict the outcome of interbacterial competitions. BW (i) detected all putative AMPs embedded in curated proteins from *UniProt*-derived bacterial proteomes, (ii) clustered the detected AMPs by sequence similarity (iii) created a database of bacterial taxa based on AMP sequence similarities and finally (iv) predicted specific taxa that were more likely to “die” in a given microbiome. The final step was achieved using a novel network-based scoring scheme (termed “*Die score*”) that provided a measure of how likely a given microbial genus/species was to “die” in a given niche. It should be noted that the *Die* score is a network measure that may not be restricted to this work. It can be applied to any network that may follow similar assumptions to the bacterial networks generated from the BW method (e.g., co-occurrence networks). The methodology was used to provide evidence in favor of the putative AMP defense hypothesis using eleven 16S rRNA gene datasets as well as one full shotgun metagenomics dataset from the gut microbiome, where the outcomes of the BW method were compared to co-occurrence networks derived from these data. In almost all examined cases, bacterial predominance seemed to be affected by the similarity of encoded AMPs in bacterial proteins.

Co-occurrence networks are subject to change depending on the parameters used during the algorithm employed for their construction (i.e., the choice of correlation metric, the *p*-value cut-off etc.). This directly impinges on the correlation scores between Die scores for BW and MB networks and although we have tried to be as judicious as possible in the use of these parameters across the twelve datasets assessed in this study, there is still an aspect of subjectivity in the use of these parameters that could potentially affect results. Another concern is the exclusive use of *AMPA* as a detector of AMPs. It could well be that not all AMPs are detectable by *AMPA* and not all AMPs predicted by *AMPA* may have antibacterial properties. All these concerns must be addressed experimentally, which is not a trivial task. We can only state that under the current handling and analysis of proteomes, AMPs and bacterial abundances, putative AMPs seem like a significant factor in the interaction of different bacteria in a chemosphere (putative AMP defense hypothesis).

Numerous databases describe AMPs from prokaryotic and eukaryotic organisms (Seshadri Sundararajan et al., 2012; Gogoladze et al., 2014; Fan et al., 2016; Lin et al., 2016; Pirtskhalava et al., 2016; Waghu et al., 2016; Wang et al., 2016). They host experimentally validated, as well as computationally predicted, AMPs and are fully searchable allowing for queries of multiple search criteria. However, they do not provide any information on the relationship among the different AMPs or some background to explain their efficiency against specific organisms. The herein developed database combined with the BW methodology has the potential of detecting relationships between AMPs in the proteomes of different bacterial taxa and provide such information for further experimentation purposes. Predictions on the predominance and outlook of specific taxa in a given microbiome may lie within the potential of the method.

A first glance on bacterial genome size and AMP count suggests that the two are not related (Figure 1A). The aphid dwelling *Buchnera aphidicola* with a relatively small genome appears as having the highest number of putative AMPs followed by *Wigglesworthia glossinidia*, that lives in the gut of tsetse flies and is considered as having one of the smallest known genomes of any living organism. On the other hand, the predatory *Bdellovibrio bacteriovorus*, and *Fusobacterium nucleatum* of the oral cavity, seem as having less putative AMPs. *E. coli* lab strain K12 has a large number of putative AMPs (Figure 1B) but relatively small compared to its proteome (Figure 1A). Could it be that bacteria that live in relatively host-independent conditions (more open chemospheres) may need less AMPs? The relationship between, abiotic conditions of living, surrounding microbiomes and hosts are some of the issues that could be the object of a comparative study with the aim of explaining the different numbers of AMPs in different bacterial taxa.

A large proportion of the putative AMPs were unique as they shared low sequence similarity (<80%) (Figure 1C). This could be viewed as the extreme versatility of the combinatorial capabilities of microorganisms to create diverse AMPs to face an opponent species. Comparison of the *Die* scores in bacteria with close phylogenetic origin showed that putative AMPs were specific to the level of genera (Figure 6).

In summary, we propose that putative AMPs hidden within the proteome of bacteria may affect their symbiosis with other species in a given chemosphere. In support of the putative AMP defense hypothesis, we provide computational data corroborated by gut microbiome datasets on the abundance of bacterial species in the enterobiomes of different diseases. We believe that our findings may add another jigsaw in the mosaic of bacterial interactions and could perhaps provide leads for the construction of species-specific AMPs.

## Materials and Methods

### Datasets

16S rRNA gene processed OTU tables were used. These represent the abundances (in counts) of specific taxa in eleven gut microbiome datasets. The datasets were downloaded from the MicrobiomeHD database (Duvallet et al., 2017), https://zenodo.org/record/569601#.XdPrYmgzaUk.

The datasets included:

1. EDD_Singh, 2015 enteric diarrheal disease (EDD) – (Singh et al., 2015).
2. CDI_Schubert, 2014 *Clostridium difficile* infection (CDI) – (Schubert et al., 2014).
3. OB_Zupancic, 2012 Obesity (OB) (Zupancic et al., 2012).
4. HIV_Noguera-Julian, 2016 HIV (Noguera-Julian et al., 2016).
5. HIV_Dinh, 2014 HIV (Dinh et al., 2015).
6. MHE_Zhang, 2013 liver diseases (LIV) (Zhang et al., 2013).
7. PAR_Scheperjans, 2014 Parkinson’s (PAR) (Scheperjans et al., 2015).
8. ASD_KB, 2013 Autism (ASD) (Kang et al., 2013).
9. ASD_Son, 2015 Autism (ASD) (Son et al., 2015).
10. NASH_Chang, 2013, non-alcoholic steatohepatitis (NASH) (Wong et al., 2013).
11. NASH_OB_Baker, 2013, non-alcoholic steatohepatitis (NASH) (Zhu et al., 2013).

Shotgun metagenomics dataset:

1. Parkinson’s disease vs. control metagenomics dataset (Becker et al., 2021).

### Predicting Antimicrobial Peptides Using *AMPA*

*AMPA* (Torrent et al., 2009, 2012), is a publicly available tool for prediction of antimicrobial peptides in a given protein sequence. It is available as a web browser^1^ but also as a standalone tool. We downloaded and utilized the source code for *AMPA* written in PERL and available under Linux, in order to perform batch runs for our proteins of interests as detailed in Figure 2. We ran *AMPA* using the default settings for all input protein sequences.

### Clustering Antimicrobial Peptides Using a Similarity Threshold

*CdHit* was used to cluster AMPs by applying a sequence similarity threshold. AMP similarity was set up to support the hypothesis that bacterial species that share common AMPs (sequence similarity >80%) may coexist more harmoniously in comparison to species that share less or no sequence similarity in their AMPs.

### Network Construction

Two different types of networks were constructed using: (i) the BW database (available as an edge list – see Supplementary Table 2) and (ii) MB abundance tables. Network constructions and co-occurrence calculations were performed in R using the following library packages: *phyloseq, vegan, ape, WGCNA, igraph*, and *network*. All networks for the 16S data were constructed at the genus level, while the network constructed for the shotgun metagenomics dataset depicts species level associations All networks generated for the BW methodology can be found as Supplementary Figures 7–10. The networks at the genus level were constructed by aggregating all the AMPs found in the species belonging to a specific genus.

### *Die* Score

The *Die* score provides a measure of how likely a particular bacterial node (genus/species) is to “die” in a given microbiome environment characterized by a network. It can also be considered as a projection of the relative abundance of bacterial taxa based on their topology in a network. The putative AMP defense hypothesis assumes that bacteria’s capacity to kill each other is inversely dependent on the similarity of their peptides. Therefore, for the BW approach, we created a graph *BW* with nodes representing different bacterial taxa and edges representing their common peptides.

For a node *i* in a given graph *BW*, we calculated the *Die* score (*D*) with equation (1):


(1) $$D_{i}=\frac{N_{i}-S_{i}}{N_{p}}$$

Where: *S*_*i*_ represents the strength of the node (i.e., the weighted degree of the node *i*) and sums up the number of neighboring bacteria that node *i* is linked to via an edge (e.g., shares common peptides), as well as the edge weight (e.g., number of common peptides). *N*_*i*_ represents the number of pairs with no edges (e.g., no common peptides). It is calculated using the expression *N*_*i*_ = *N*_*p*_ − deg_*i*_, where deg_*i*_ represents the degree of the node *i*. *N*_*p*_ = (*n*^2^ − *n*)/2 represents the number of all possible pairs in the network.

The *Die* score can take values in the range of [−1, 1]. A value of 1 means that a particular bacterial taxon (genus/species) is more likely to die, while a value of −1 means exactly the opposite (less likely to die). After the initial calculation using eq. (1), the *Die* score was normalized by extracting the mean and dividing by the standard deviation. This allowed for a greater range for the *Die* score for better highlighting differences among the taxa analyzed by this measure.

The *Die* score formula described above, can also be applied to microbiome (*MB*) co-occurrence networks (or graphs). In this case, instead of common AMPs, the edges represent the relative abundances of the particular bacterial taxa (represented as nodes). The co-occurrence networks have to be modified to bi-directed networks, whereby the outgoing edge weight represents the relative abundance of the specific node under consideration.

### *Dist* Measure

The *Dist* measure was given by the following eq. (2):


(2) $$Dist=\left|RNK\left(Die_{BW}\right)-RNK\left(Die_{MB}\right)\right|$$

*RNK* is the ranking function performed, whereby all taxa are ranked according to their position in the *Die* score vector (the genus/species with the lowest *Die* was ranked as 1, and increasing rankings denote higher *Die* scores). The R function named “*rank(),”* was used to rank the *Die* scores. In case of ranking ties, the average of the rankings was calculated, accounting for some decimal places in the ranking tables (as shown in Supplementary Table 3). *Dist* is calculated as the absolute difference between the ranked *Die* scores from the BW and MB networks, *Die*_*BW*_ and *Die*_*MB*_, respectively.

### Skewness

Skewness was calculated using the R function “*skewness()*” in the e1071 package, according to Joanes and Gill (1998). The equation used is: *G_1 = g_1 ^∗^ sqrt(n(n-1)/(n-2).* Positive skewness was determined by any one of the following observations: histograms showing a short left tail and a long right tail, skewness calculations with value >0.4 and/or median of the distribution being closer to the first quartile – as depicted in the boxplots.

## Data Availability Statement

Publicly available datasets were analyzed in this study. This data can be found here: https://zenodo.org/record/569601#.XdPrYmgzaUk, https://www.medrxiv.org/content/10.1101/2021.02.07.21251098v1.

## Author Contributions

AO, MZ, and GMS designed the study. AO and MZ conceived and developed the computational tools. AO, CC, and MT analyzed the data and interpreted the results. GMS and AV-G interpreted the results. UI contributed to code for the computational tools. GPS contributed to the Parkinson’s metagenome dataset. AO and AV-G designed the experiments and wrote the manuscript. AV-G conceived the project. All authors edited the manuscript.

## Conflict of Interest

The authors declare that the research was conducted in the absence of any commercial or financial relationships that could be construed as a potential conflict of interest.

## Publisher’s Note

All claims expressed in this article are solely those of the authors and do not necessarily represent those of their affiliated organizations, or those of the publisher, the editors and the reviewers. Any product that may be evaluated in this article, or claim that may be made by its manufacturer, is not guaranteed or endorsed by the publisher.

## Footnotes

1
http://tcoffee.crg.cat/apps/ampa/do
