# Supplementary material for: Putative Antimicrobial Peptides Within Bacterial Proteomes Affect Bacterial Predominance: A Network Analysis Perspective
Source: Front Microbiol. 2021 Nov 12;12:752674. doi: 10.3389/fmicb.2021.752674 (PMC8636115; doi:10.3389/fmicb.2021.752674)
Supplement: Supplementary file 1 [file Data_Sheet_1.docx]

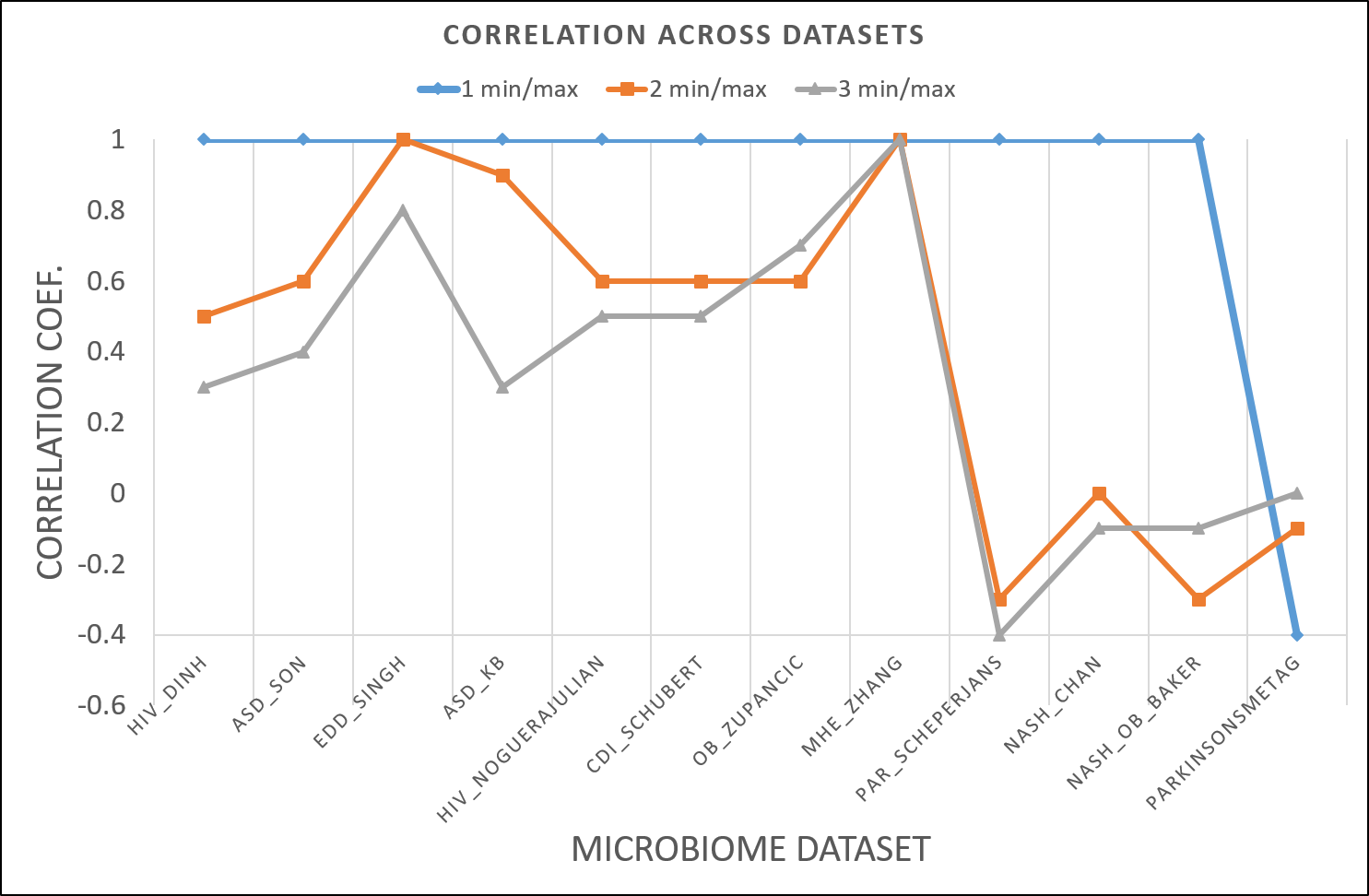


**Figure S1**. Correlation of *Die* scores for BW methodology and microbiome abundance data across 12 datasets. The blue line shows the correlation obtained when comparing taxa with the lowest and highest (1 min/max) *Die* scores from the BW network, to the *Die* scores from the corresponding taxa obtained from the MB network. The dark orange line shows the correlation coefficient when taxa with the second lowest and highest *Die* scores (2 min/max) were included in the comparison. The grey line shows the correlation results when taxa with third lowest and highest *Die* scores (3 min/max) values were also included in the comparison between networks.


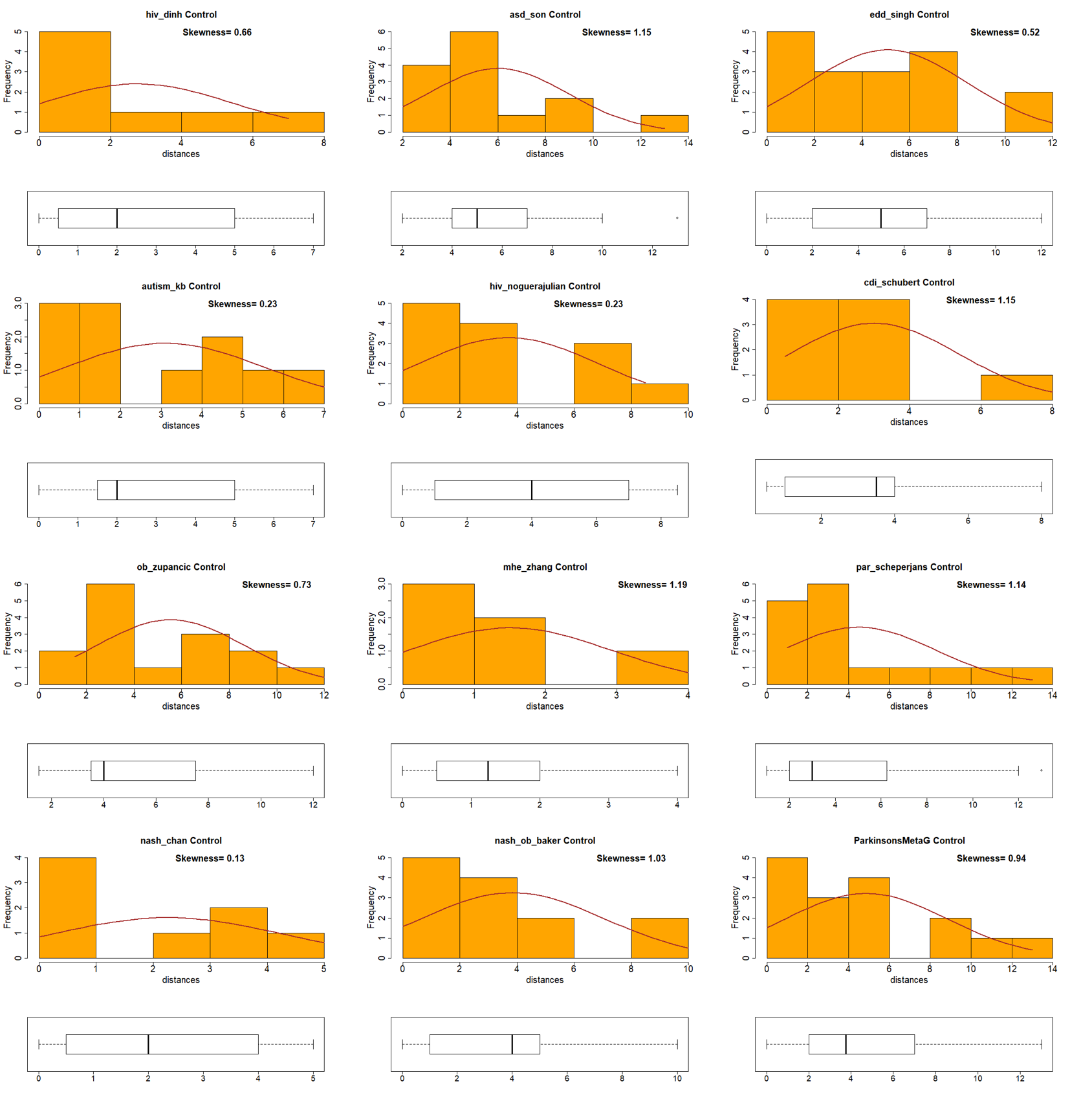


**Figure S2**. Distribution of skewness and distances (*Dist*) of the microbiomes from the 12 healthy state controls. Positively skewed distributions of *Dist* measures were observed for all 12 microbiomes.


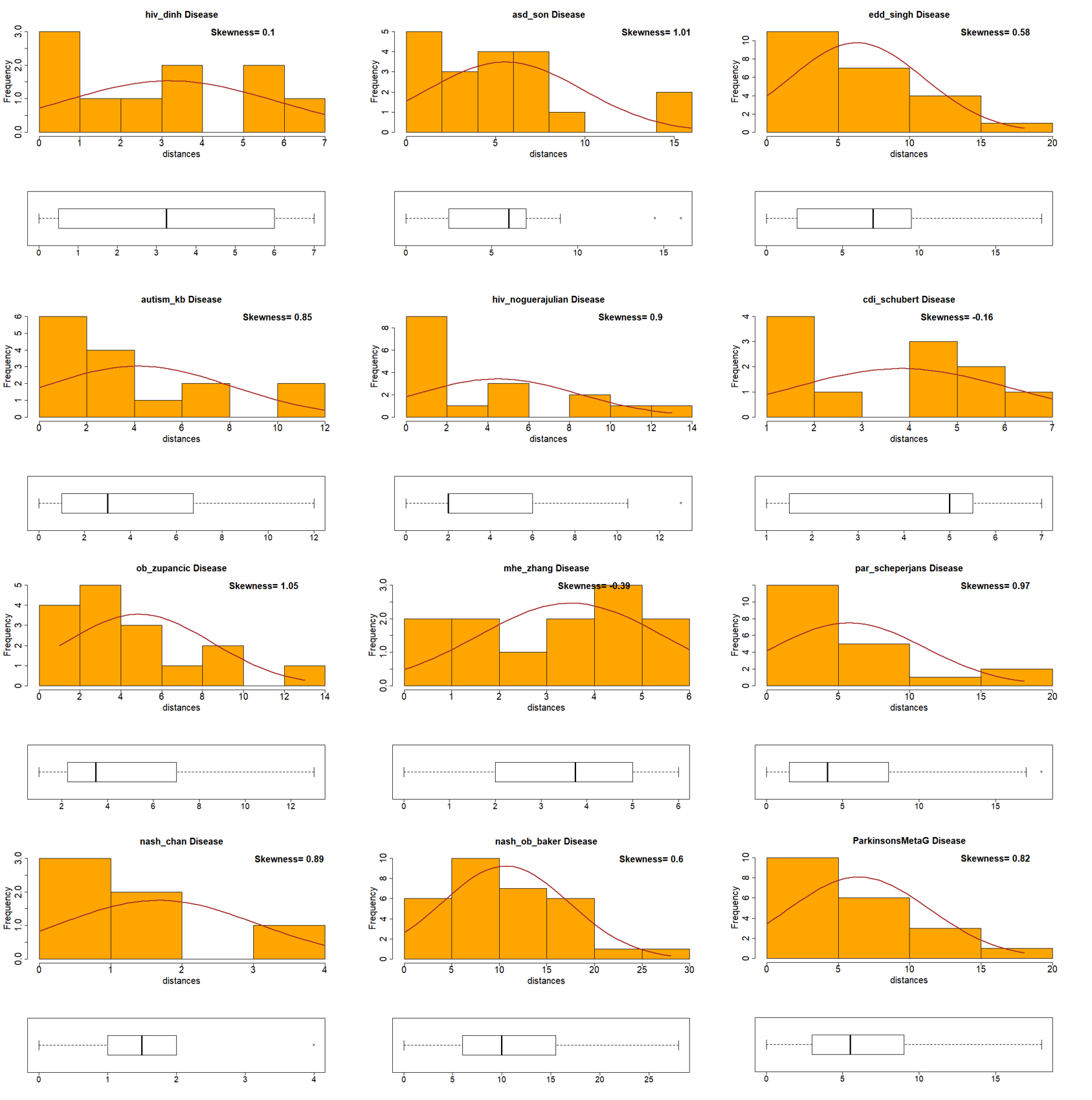


**Figure S3**. Distribution of skewness and distances (*Dist*) across 12 microbiomes from the diseased-state samples. Positively skewed distributions of *Dist* measures were observed for 8 out of 12 microbiomes.


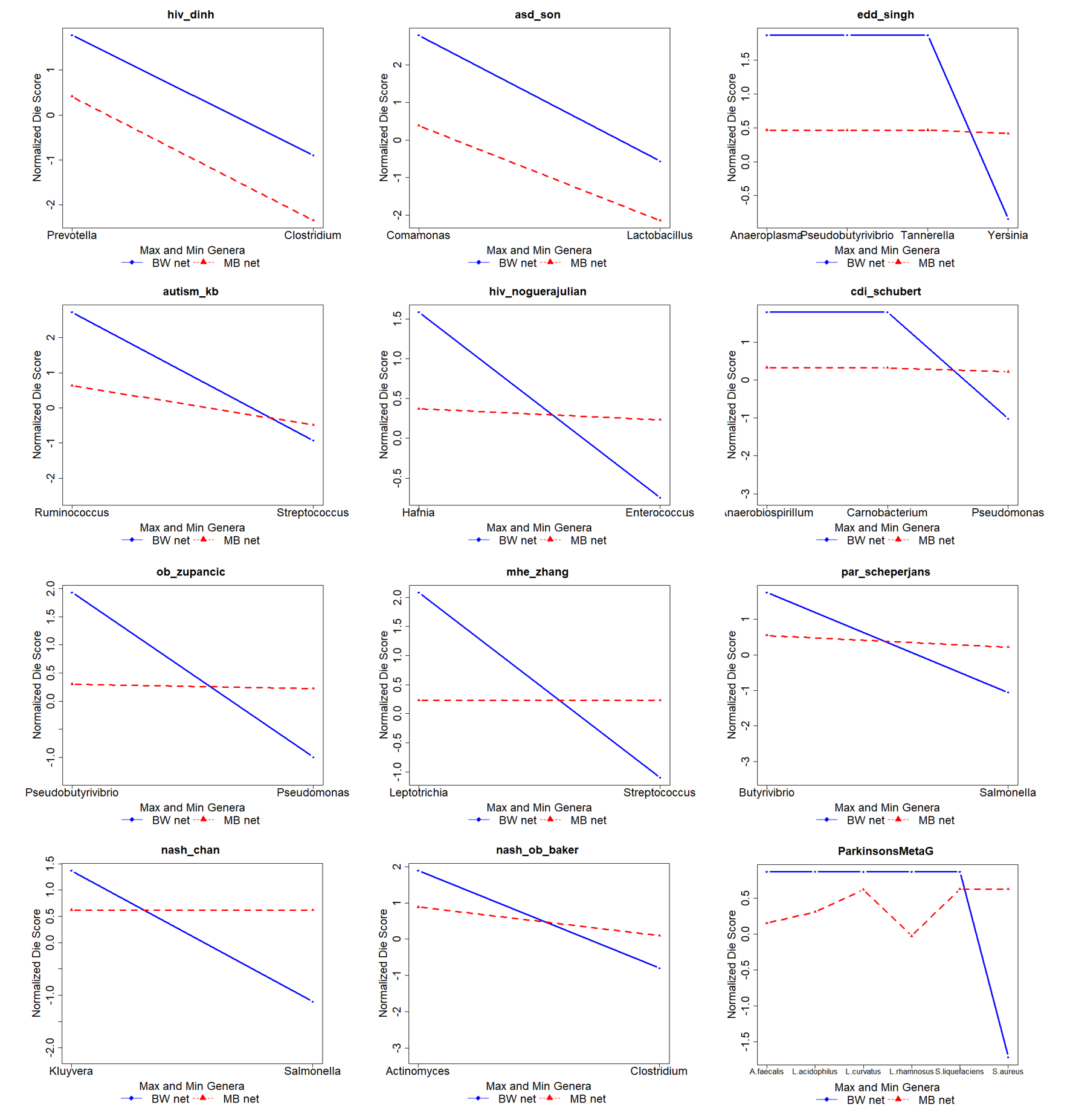


**Figure S4**. Correlation line plots showing the normalized *Die* scores for the maximum (Max) and minimum (Min) genera across all 12 datasets. Plots were obtained by pooling together diseased and healthy state microbiomes.


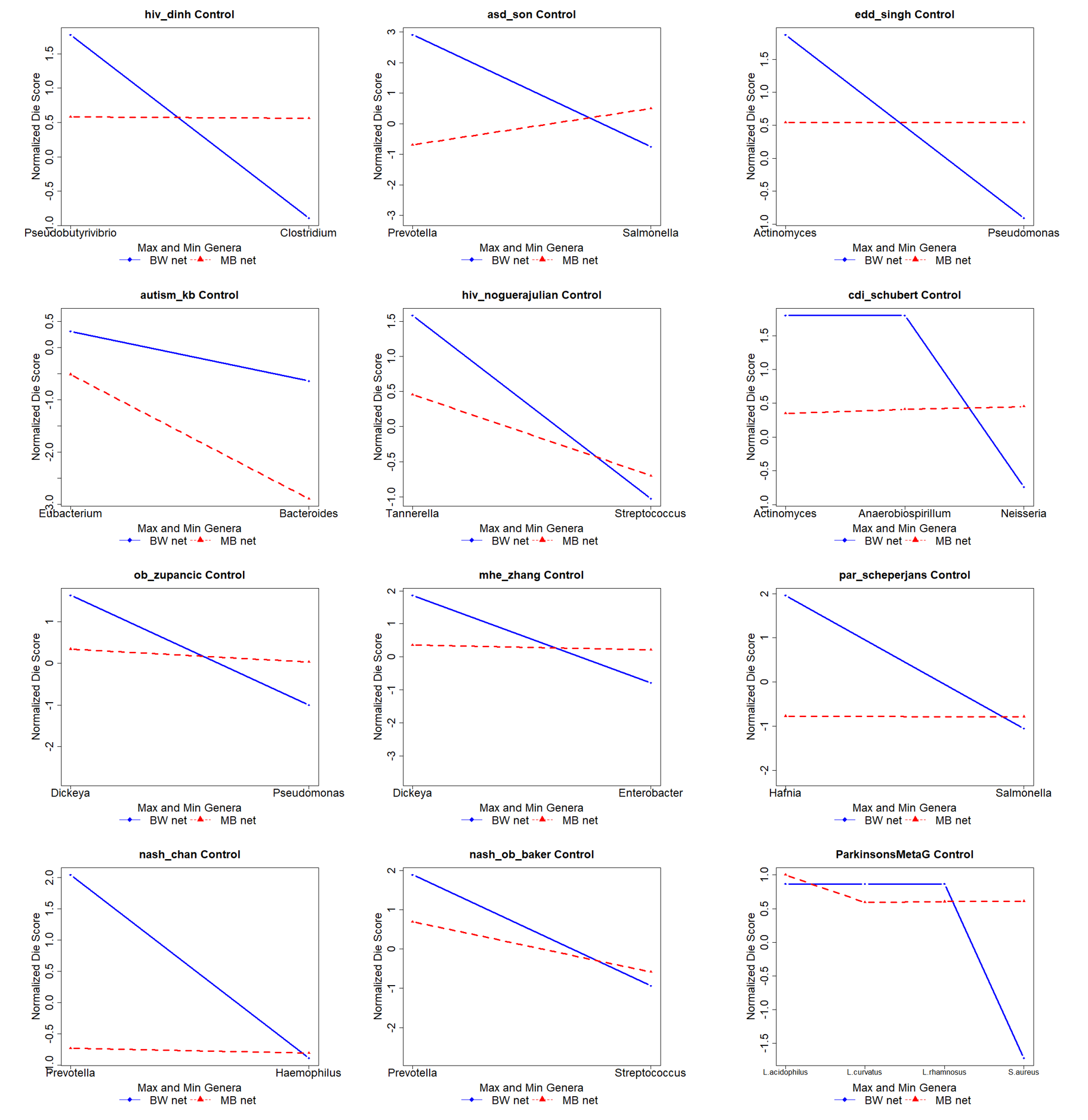


**Figure S5**. Correlation line plots showing the normalized *Die* scores for the maximum (Max) and minimum (Min) genera across all 12 datasets. Plots were obtained by analysing the healthy state samples.


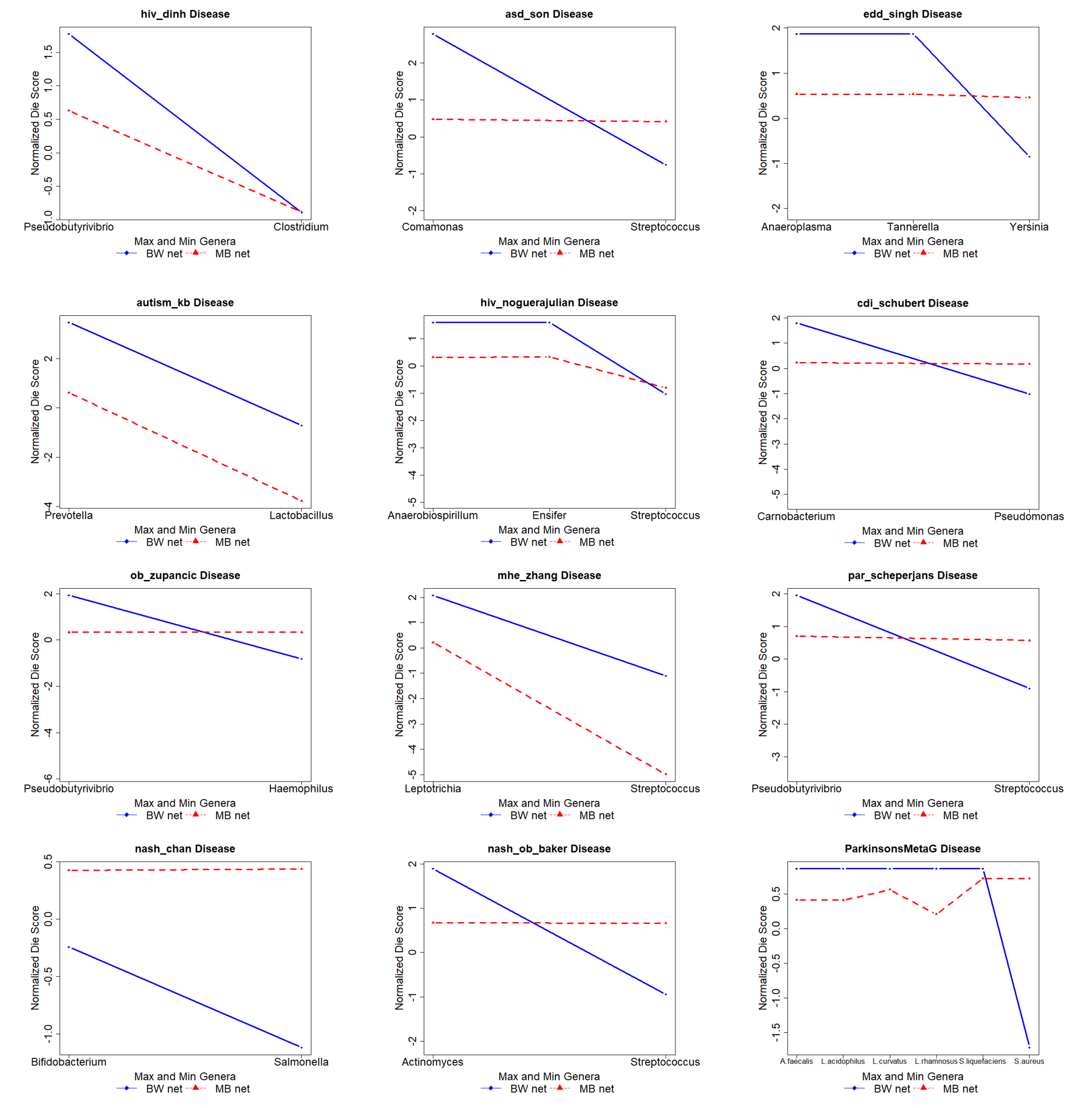


**Figure S6**. Correlation line plots showing the normalized *Die* scores for the maximum (Max) and minimum (Min) genera across all 12 datasets. Plots were obtained by analysing the diseased state samples.


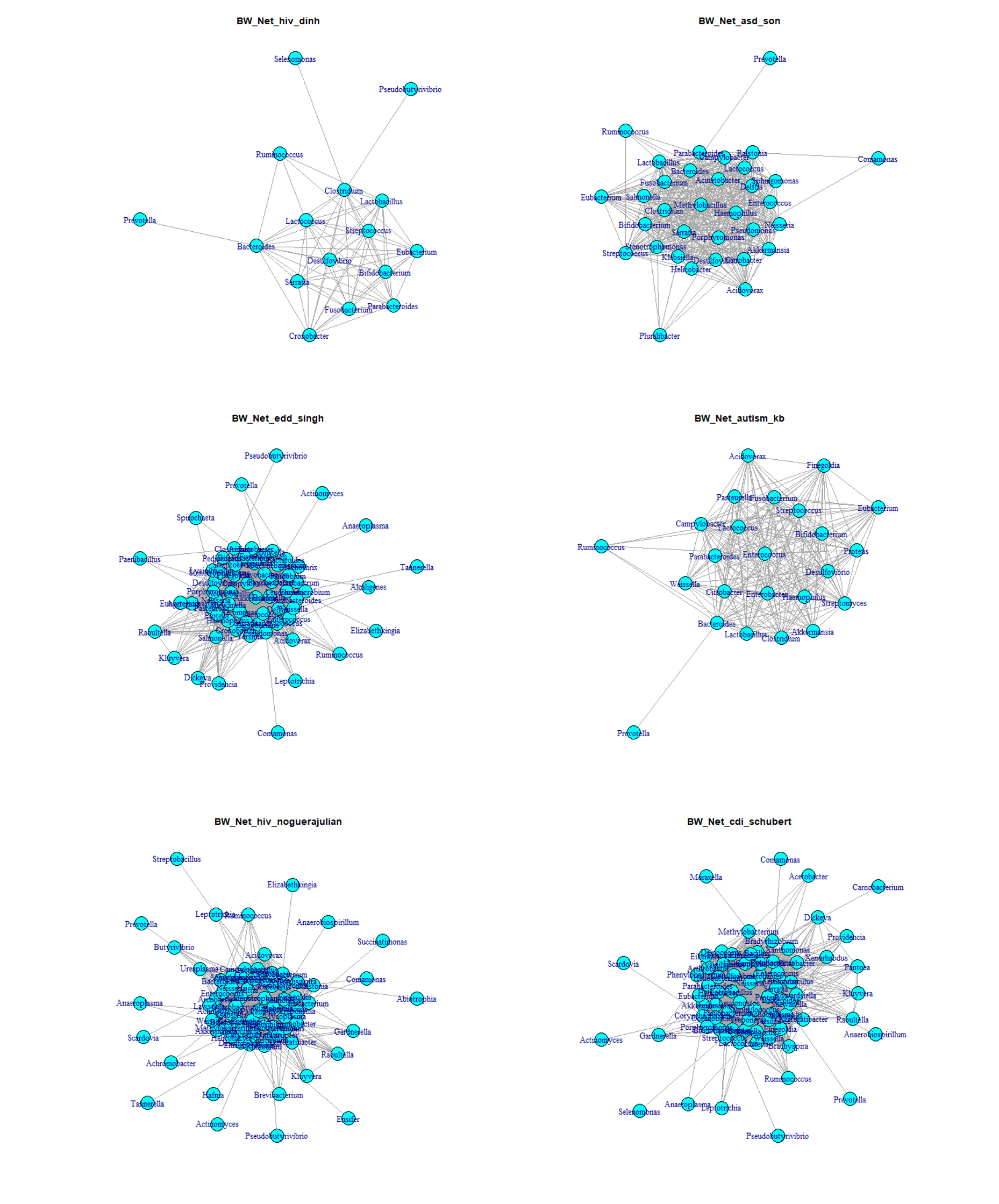

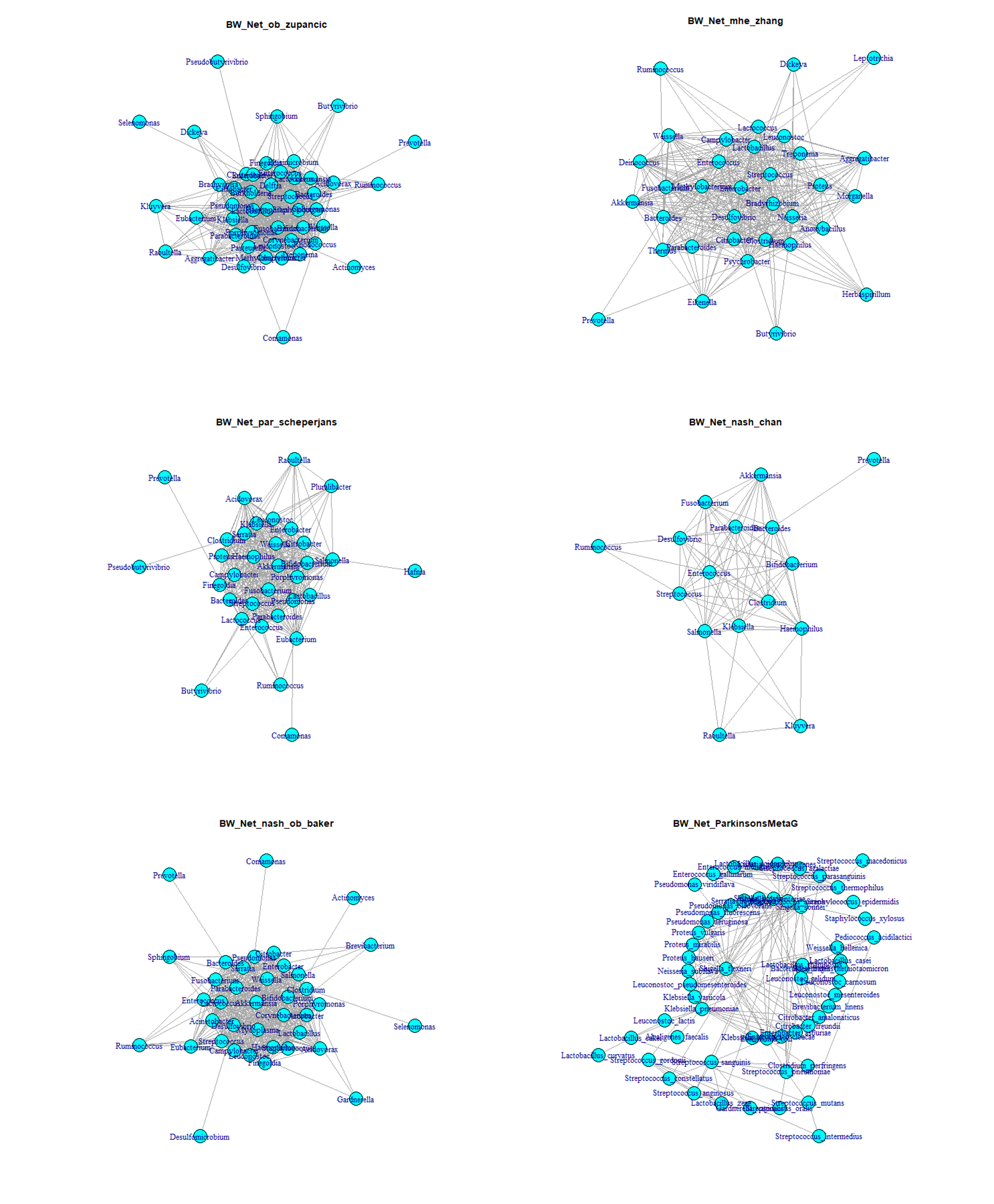


**Figure S7**. Bacterial Wars (BW) networks obtained by analysing all 12 microbiome datasets pooling together diseased and healthy state controls. Nodes represent individual genera while the edges are the number of common peptides between genera.


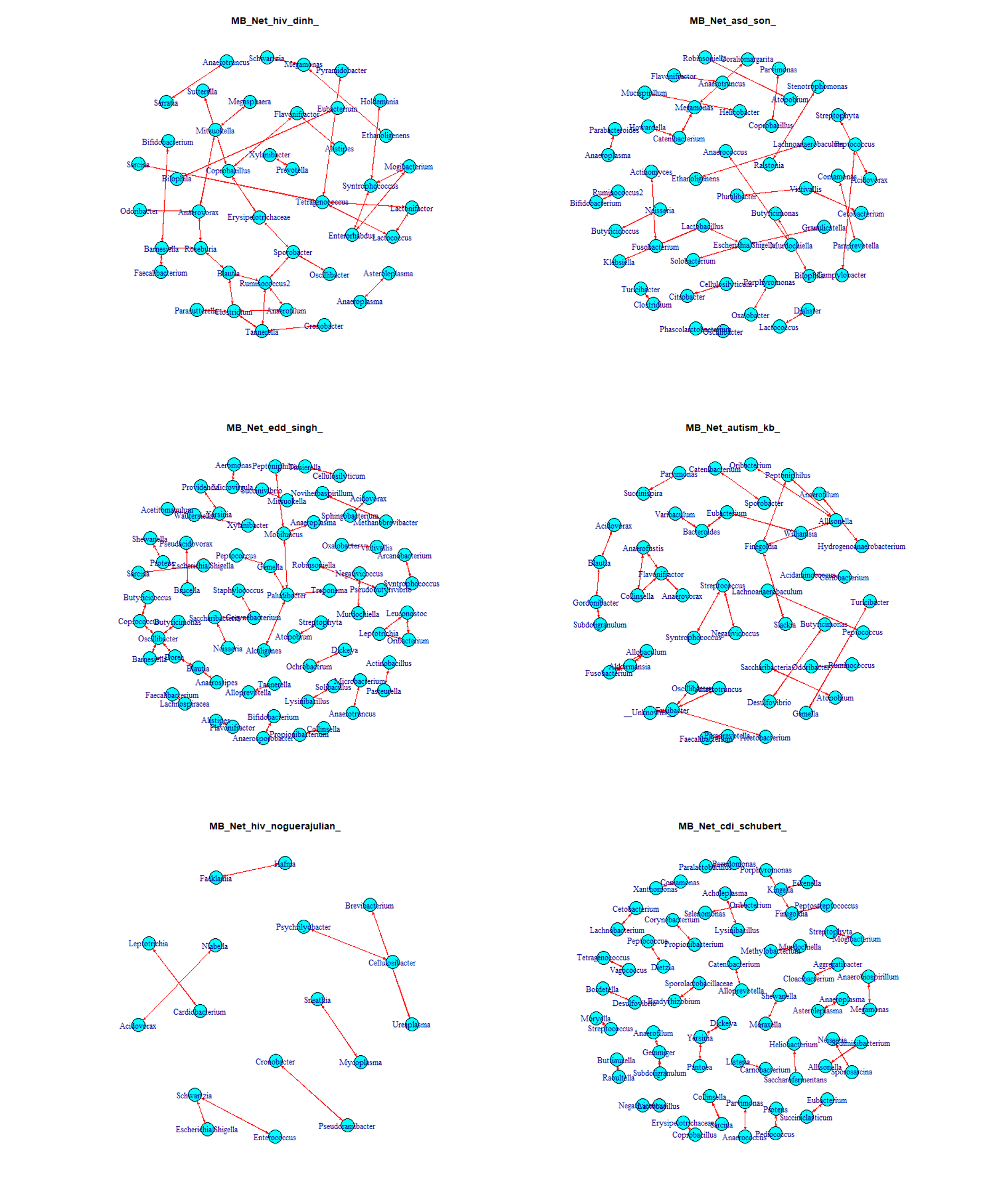

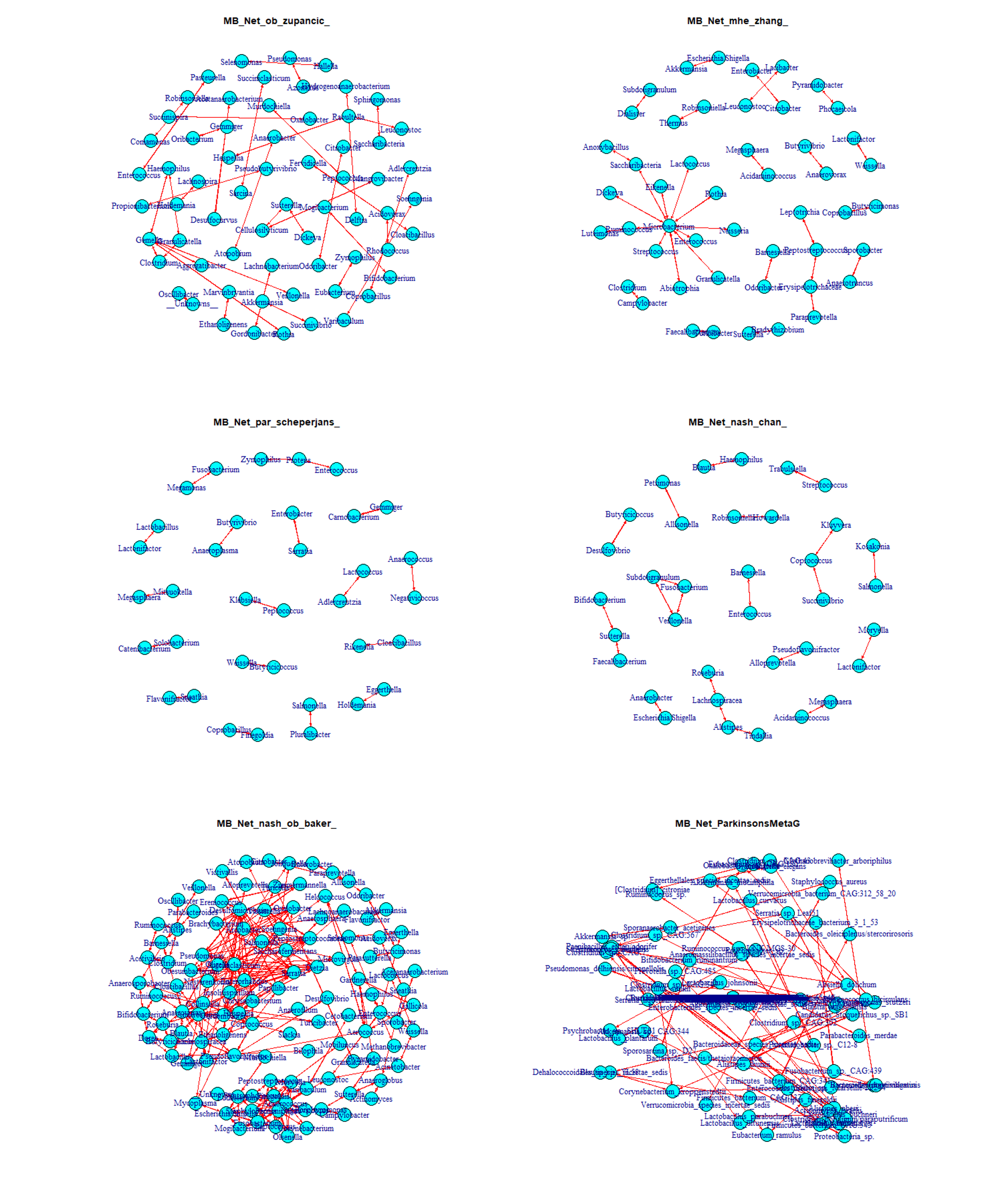


**Figure S8**. Microbiome (MB) networks obtained by analysing all 12 datasets. These networks are bi-directed co-occurrence networks obtained by pooling both diseased states and healthy state controls together. Nodes represent individual genera while the edges are bi-directed, where each outgoing edge (arrow leaving a node) shows the abundance of each node in the given microbiome.


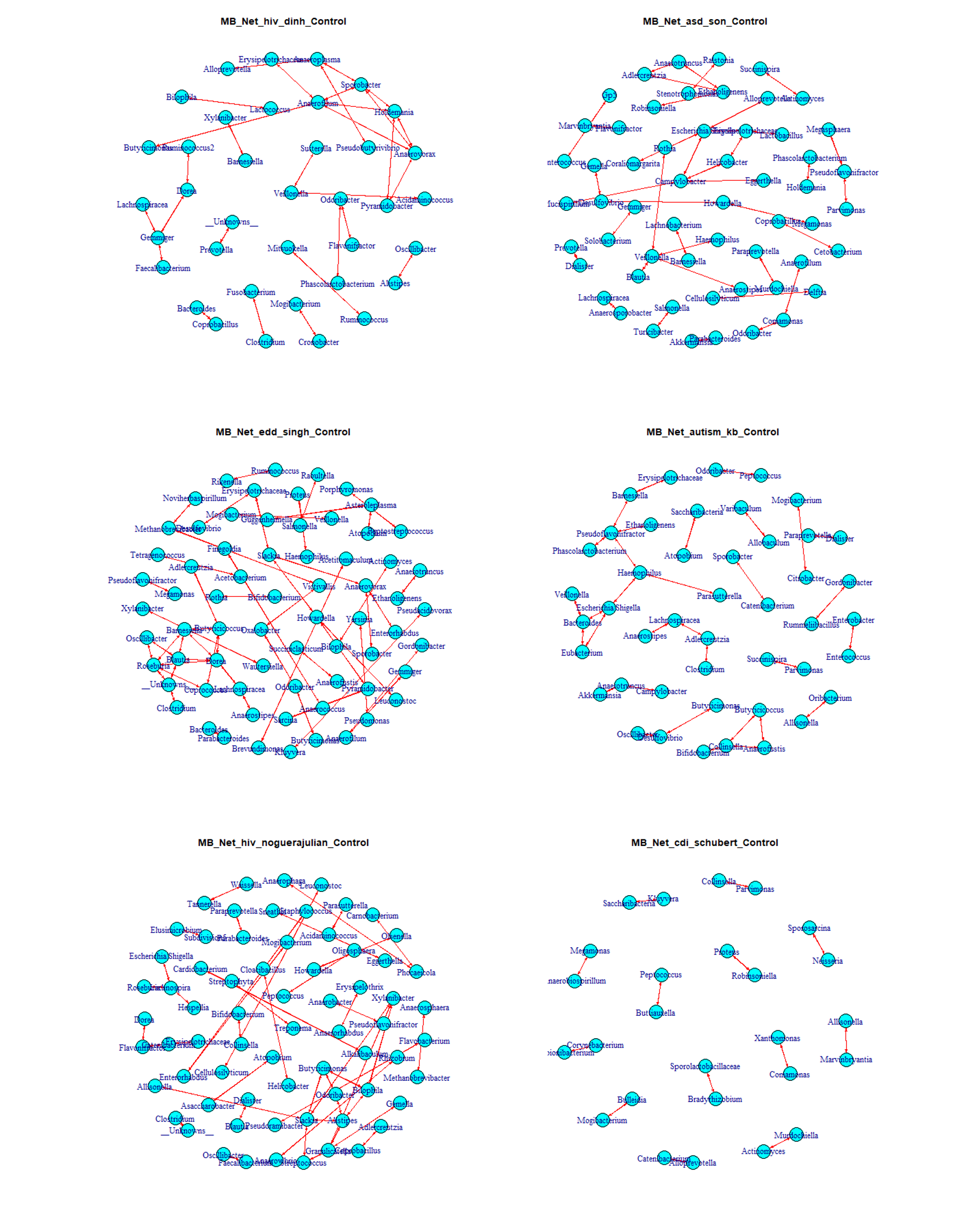

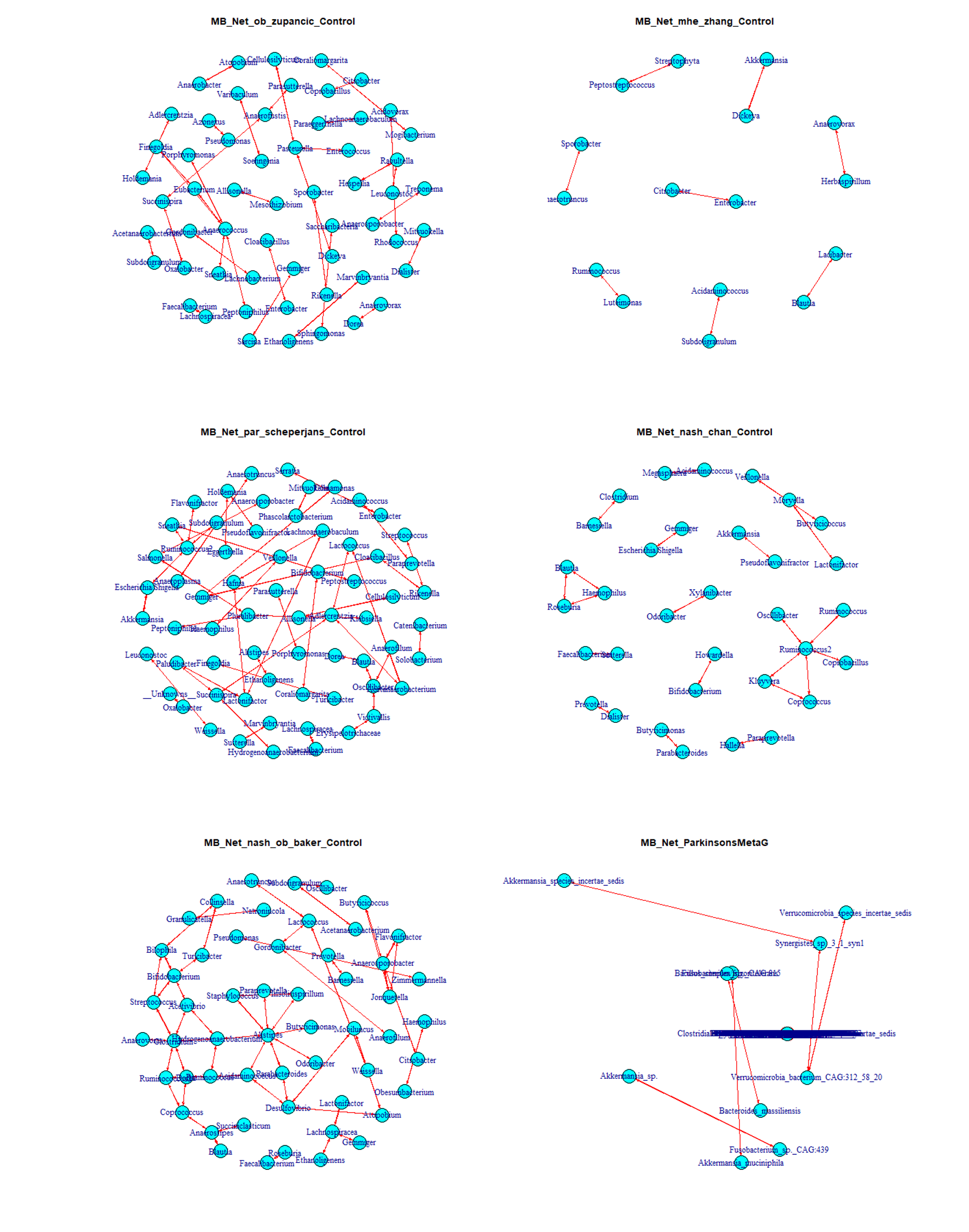


**Figure S9**. Microbiome (MB) networks obtained by analysing all 12 datasets. These networks are co-occurrence networks and they were obtained by analysing the healthy state controls.


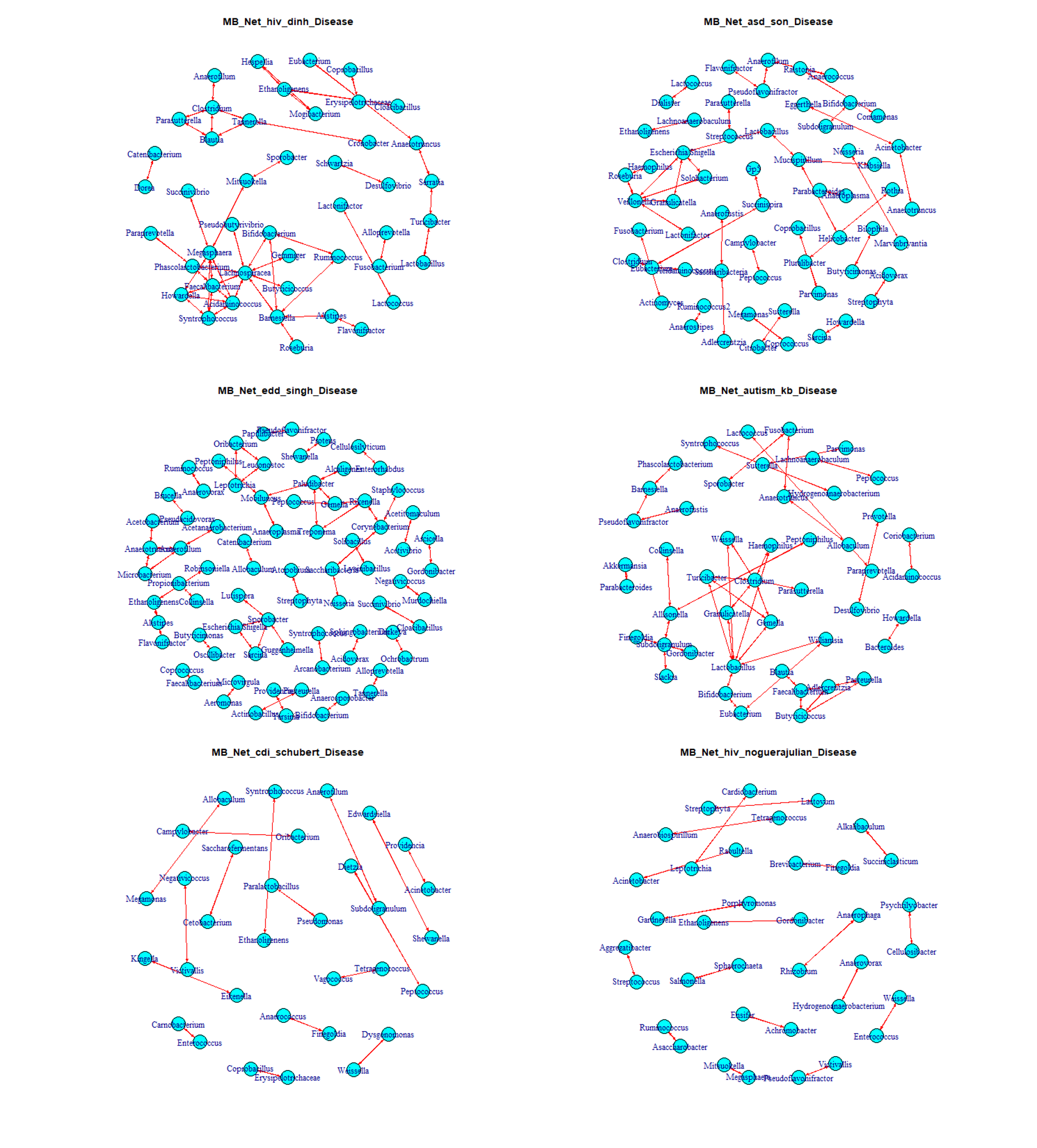

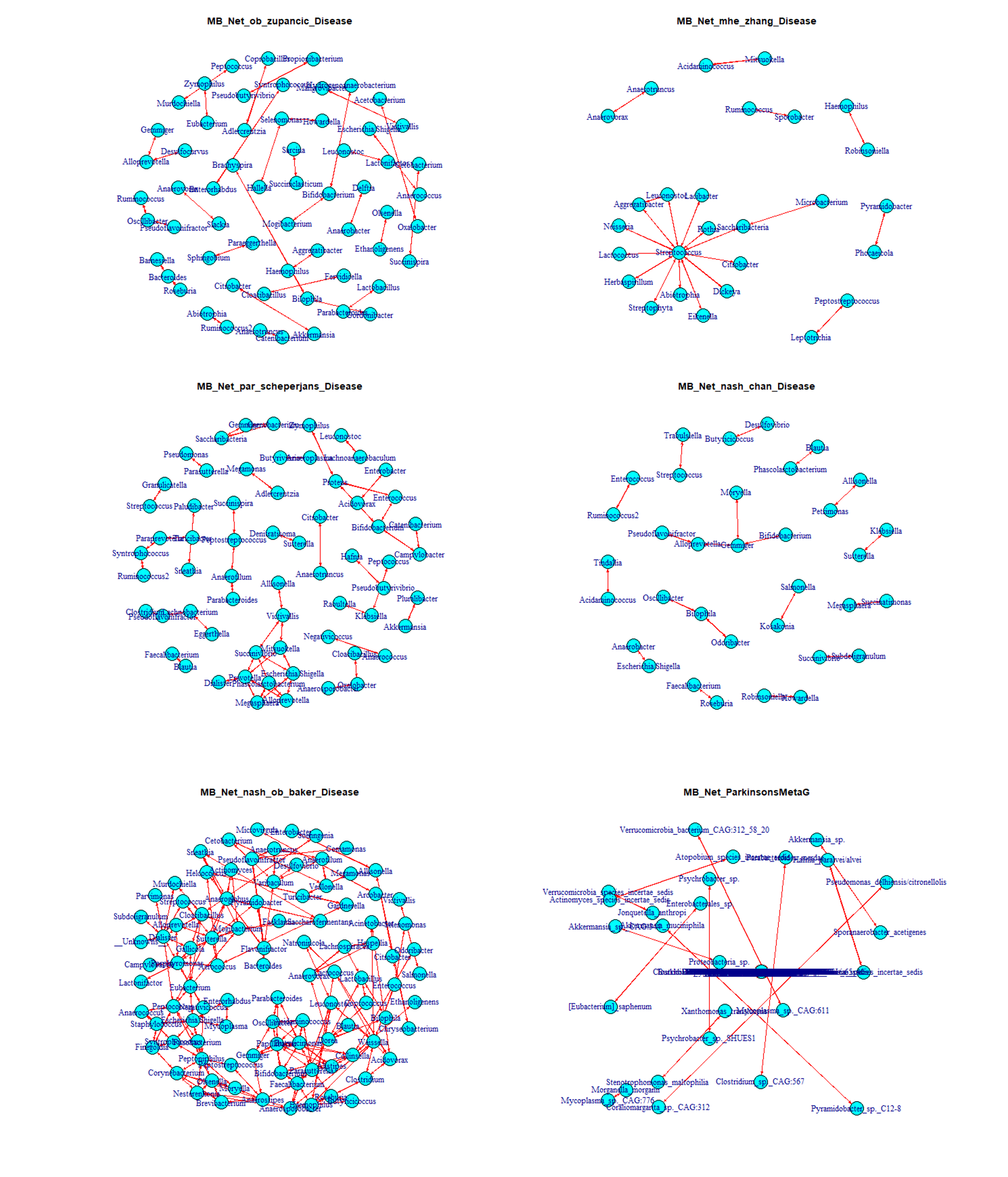


**Figure S10**. Microbiome (MB) networks obtained by analysing all 12 datasets. These networks are co-occurrence networks and they were obtained by analysing data from the 12 diseased states.

**Table S3**. Table of *Die* and *Dist* (distances) score calculation for pooled healthy controls and diseased state samples across 12 datasets.

| hiv_dinh |  |  |  |  |  |
| --- | --- | --- | --- | --- | --- |
| Genera | Die_BW | Die_MB | Die_BWranked | Die_MBranked | distances |
| *Bifidobacterium* | -0.35079 | 0.864942 | 3 | 5 | 2 |
| *Clostridium* | -0.89387 | -2.34349 | 1 | 1 | 0 |
| *Cronobacter* | -0.32889 | 0.861011 | 5 | 4 | 1 |
| *Eubacterium* | -0.32541 | 0.865429 | 6 | 7 | 1 |
| *Lactococcus* | -0.65847 | -0.1008 | 2 | 2 | 0 |
| *Prevotella* | 1.77432 | 0.411515 | 7 | 3 | 4 |
| *Serratia* | -0.33037 | 0.865241 | 4 | 6 | 2 |
|  |  |  |  |  |  |
| asd_son |  |  |  |  |  |
| Genera | Die_BW | Die_MB | Die_BWranked | Die_MBranked | distances |
| *Acidovorax* | -0.17002 | 0.382912 | 15 | 14.5 | 0.5 |
| *Bifidobacterium* | -0.28478 | 0.286945 | 10 | 4 | 6 |
| *Campylobacter* | -0.30919 | 0.380601 | 8 | 7 | 1 |
| *Citrobacter* | -0.44306 | 0.378695 | 4 | 6 | 2 |
| *Clostridium* | -0.38986 | -2.11026 | 6 | 2 | 4 |
| *Comamonas* | 2.788856 | 0.38297 | 17 | 16.5 | 0.5 |
| *Fusobacterium* | -0.26111 | 0.382508 | 14 | 12 | 2 |
| *Helicobacter* | -0.30599 | 0.38245 | 9 | 11 | 2 |
| *Klebsiella* | -0.50046 | 0.38297 | 2 | 16.5 | 14.5 |
| *Lactobacillus* | -0.56456 | -2.13753 | 1 | 1 | 0 |
| *Lactococcus* | -0.45215 | 0.374361 | 3 | 5 | 2 |
| *Neisseria* | -0.33391 | 0.382912 | 7 | 14.5 | 7.5 |
| *Parabacteroides* | -0.26136 | -0.13176 | 13 | 3 | 10 |
| *Pluralibacter* | 2.203671 | 0.381121 | 16 | 8 | 8 |
| *Porphyromonas* | -0.26813 | 0.382161 | 12 | 10 | 2 |
| *Ralstonia* | -0.39974 | 0.382046 | 5 | 9 | 4 |
| *Stenotrophomonas* | -0.27483 | 0.382855 | 11 | 13 | 2 |
|  |  |  |  |  |  |
| edd_singh | |  |  |  |  |
| Genera | Die_BW | Die_MB | Die_BWranked | Die_MBranked | distances |
| *Acidovorax* | -0.41157 | 0.46472 | 16 | 14 | 2 |
| *Actinobacillus* | -0.75167 | 0.458896 | 3 | 11 | 8 |
| *Aeromonas* | -0.68573 | 0.363465 | 4 | 4 | 0 |
| *Alcaligenes* | 1.764953 | 0.46609 | 20 | 18 | 2 |
| *Anaeroplasma* | 1.870618 | 0.466699 | 22 | 23 | 1 |
| *Bifidobacterium* | -0.5167 | 0.40549 | 11 | 6 | 5 |
| *Brucella* | -0.53191 | 0.466547 | 10 | 21 | 11 |
| *Corynebacterium* | -0.6289 | -0.86518 | 8 | 3 | 5 |
| *Dickeya* | 1.288923 | 0.466661 | 17 | 22 | 5 |
| *Leptotrichia* | 1.71199 | -0.86822 | 19 | 1 | 18 |
| *Leuconostoc* | -0.51392 | -0.86571 | 12 | 2 | 10 |
| *Lysinibacillus* | -0.51107 | 0.465976 | 14 | 17 | 3 |
| *Neisseria* | -0.63721 | 0.462664 | 7 | 13 | 6 |
| *Ochrobactrum* | -0.51246 | 0.460304 | 13 | 12 | 1 |
| *Pasteurella* | -0.67785 | 0.465862 | 6 | 15 | 9 |
| *Proteus* | -0.57283 | 0.449874 | 9 | 9 | 0 |
| *Providencia* | 1.289276 | 0.404767 | 18 | 5 | 13 |
| *Pseudobutyrivibrio* | 1.870618 | 0.466281 | 22 | 19 | 3 |
| *Shewanella* | -0.83868 | 0.410629 | 2 | 7 | 5 |
| *Staphylococcus* | -0.67868 | 0.456802 | 5 | 10 | 5 |
| *Tannerella* | 1.870618 | 0.466471 | 22 | 20 | 2 |
| *Treponema* | -0.50961 | 0.4659 | 15 | 16 | 1 |
| *Yersinia* | -0.84979 | 0.41748 | 1 | 8 | 7 |
|  |  |  |  |  |  |
| autism_kb | |  |  |  |  |
| Genera | Die_BW | Die_MB | Die_BWranked | Die_MBranked | distances |
| *Acidovorax* | -0.07923 | 0.658796 | 6 | 9 | 3 |
| *Akkermansia* | 0.123079 | -2.55574 | 7 | 1 | 6 |
| *Bacteroides* | -0.6447 | -0.78476 | 2 | 3 | 1 |
| *Desulfovibrio* | -0.29141 | 0.65585 | 3 | 8 | 5 |
| *Eubacterium* | 0.306588 | -0.45848 | 8 | 5 | 3 |
| *Finegoldia* | -0.25665 | -1.56893 | 5 | 2 | 3 |
| *Fusobacterium* | -0.25712 | -0.45459 | 4 | 6 | 2 |
| *Ruminococcus* | 2.729378 | 0.63144 | 9 | 7 | 2 |
| *Streptococcus* | -0.92823 | -0.48637 | 1 | 4 | 3 |
|  |  |  |  |  |  |
| hiv_noguerajulian | |  |  |  |  |
| Genera | Die_BW | Die_MB | Die_BWranked | Die_MBranked | distances |
| *Acidovorax* | -0.51972 | 0.369783 | 4 | 5.5 | 1.5 |
| *Brevibacterium* | 1.405225 | 0.369783 | 7 | 5.5 | 1.5 |
| *Cronobacter* | -0.70708 | 0.369783 | 2 | 5.5 | 3.5 |
| *Enterococcus* | -0.74683 | 0.23277 | 1 | 1 | 0 |
| *Hafnia* | 1.587263 | 0.369901 | 8 | 8 | 0 |
| *Leptotrichia* | 1.405087 | 0.369427 | 6 | 3 | 3 |
| *Mycoplasma* | -0.66625 | 0.362428 | 3 | 2 | 1 |
| *Ureaplasma* | 0.220536 | 0.369783 | 5 | 5.5 | 0.5 |
|  |  |  |  |  |  |
| cdi_schubert | |  |  |  |  |
| Genera | Die_BW | Die_MB | Die_BWranked | Die_MBranked | distances |
| *Acholeplasma* | -0.62668 | 0.30387 | 12 | 12 | 0 |
| *Aggregatibacter* | -0.12262 | 0.332826 | 22 | 20.5 | 1.5 |
| *Anaerobiospirillum* | 1.799697 | 0.333691 | 31.5 | 24 | 7.5 |
| *Anaeroplasma* | 1.753954 | 0.173134 | 27 | 7 | 20 |
| *Bordetella* | -0.64536 | 0.329801 | 10 | 17 | 7 |
| *Bradyrhizobium* | -0.67857 | 0.333907 | 9 | 25.5 | 16.5 |
| *Carnobacterium* | 1.799697 | 0.324183 | 31.5 | 15 | 16.5 |
| *Comamonas* | 1.799672 | 0.330017 | 29 | 18 | 11 |
| *Corynebacterium* | -0.73216 | 0.29998 | 6 | 10 | 4 |
| *Desulfovibrio* | -0.59153 | 0.311433 | 13 | 14 | 1 |
| *Dickeya* | 1.158834 | 0.334339 | 24 | 29.5 | 5.5 |
| *Eikenella* | 0.335668 | 0.334555 | 23 | 31.5 | 8.5 |
| *Eubacterium* | -0.16812 | 0.302789 | 21 | 11 | 10 |
| *Finegoldia* | -0.4897 | -2.60041 | 20 | 2 | 18 |
| *Heliobacterium* | -0.62877 | 0.334339 | 11 | 29.5 | 18.5 |
| *Lactobacillus* | -0.68402 | -2.466 | 8 | 3 | 5 |
| *Listeria* | -0.57552 | 0.334123 | 14 | 27.5 | 13.5 |
| *Lysinibacillus* | -0.49328 | 0.327424 | 18 | 16 | 2 |
| *Methylobacterium* | -0.54404 | 0.333475 | 16 | 23 | 7 |
| *Moraxella* | 1.799677 | 0.334123 | 30 | 27.5 | 2.5 |
| *Neisseria* | -0.73895 | 0.334555 | 5 | 31.5 | 26.5 |
| *Pantoea* | 1.204852 | 0.333042 | 25 | 22 | 3 |
| *Pediococcus* | -0.53833 | 0.28507 | 17 | 9 | 8 |
| *Porphyromonas* | -0.49125 | 0.30884 | 19 | 13 | 6 |
| *Proteus* | -0.72742 | 0.132725 | 7 | 6 | 1 |
| *Pseudomonas* | -1.02073 | 0.2237 | 1 | 8 | 7 |
| *Raoultella* | 1.250508 | -2.1799 | 26 | 4 | 22 |
| *Selenomonas* | 1.79967 | 0.332826 | 28 | 20.5 | 7.5 |
| *Shewanella* | -0.95889 | 0.333907 | 3 | 25.5 | 22.5 |
| *Streptococcus* | -0.82102 | -1.45015 | 4 | 5 | 1 |
| *Xanthomonas* | -0.55481 | 0.33261 | 15 | 19 | 4 |
| *Yersinia* | -0.96768 | -3.1078 | 2 | 1 | 1 |
|  |  |  |  |  |  |
| ob_zupancic | |  |  |  |  |
| Genera | Die_BW | Die_MB | Die_BWranked | Die_MBranked | distances |
| *Acidovorax* | -0.47356 | 0.302613 | 10 | 20 | 10 |
| *Aggregatibacter* | 0.144164 | 0.30259 | 14 | 14 | 0 |
| *Akkermansia* | -0.30203 | 0.30043 | 13 | 5 | 8 |
| *Bifidobacterium* | -0.54471 | 0.302535 | 7 | 9 | 2 |
| *Citrobacter* | -0.76085 | 0.30182 | 5 | 6 | 1 |
| *Clostridium* | -0.84065 | -1.36282 | 2 | 3 | 1 |
| *Comamonas* | 1.857476 | 0.302582 | 18 | 12 | 6 |
| *Delftia* | -0.45826 | 0.302597 | 11 | 17 | 6 |
| *Dickeya* | 1.633865 | 0.302487 | 17 | 8 | 9 |
| *Enterococcus* | -0.76852 | 0.302126 | 4 | 7 | 3 |
| *Eubacterium* | 0.21848 | 0.302574 | 15 | 10.5 | 4.5 |
| *Haemophilus* | -0.80736 | -1.36452 | 3 | 1 | 2 |
| *Leuconostoc* | -0.54002 | 0.302597 | 8 | 17 | 9 |
| *Pasteurella* | -0.68791 | 0.30259 | 6 | 14 | 8 |
| *Pseudobutyrivibrio* | 1.931969 | 0.302597 | 20 | 17 | 3 |
| *Pseudomonas* | -0.99859 | 0.219906 | 1 | 4 | 3 |
| *Raoultella* | 1.410627 | -1.36288 | 16 | 2 | 14 |
| *Rhodococcus* | -0.38848 | 0.302574 | 12 | 10.5 | 1.5 |
| *Selenomonas* | 1.931883 | 0.30259 | 19 | 14 | 5 |
| *Sphingomonas* | -0.52803 | 0.302605 | 9 | 19 | 10 |
|  |  |  |  |  |  |
| mhe_zhang | |  |  |  |  |
| Genera | Die_BW | Die_MB | Die_BWranked | Die_MBranked | distances |
| *Akkermansia* | -0.01016 | 0.229104 | 14 | 15.5 | 1.5 |
| *Anoxybacillus* | -0.12582 | 0.228867 | 12 | 12.5 | 0.5 |
| *Bradyrhizobium* | -0.57957 | 0.22934 | 9 | 18.5 | 9.5 |
| *Butyrivibrio* | 1.973867 | 0.229104 | 18 | 15.5 | 2.5 |
| *Campylobacter* | -0.58479 | 0.227685 | 8 | 6 | 2 |
| *Citrobacter* | -0.68184 | 0.224137 | 7 | 4 | 3 |
| *Clostridium* | -0.77305 | -0.3523 | 5 | 2 | 3 |
| *Dickeya* | 1.863475 | 0.229104 | 17 | 15.5 | 1.5 |
| *Eikenella* | 0.872093 | 0.228394 | 15 | 9 | 6 |
| *Enterobacter* | -0.79275 | 0.225556 | 4 | 5 | 1 |
| *Enterococcus* | -0.6983 | 0.228394 | 6 | 9 | 3 |
| *Lactococcus* | -0.8526 | 0.228394 | 3 | 9 | 6 |
| *Leptotrichia* | 2.083517 | 0.228867 | 19 | 12.5 | 6.5 |
| *Leuconostoc* | -0.47035 | 0.22934 | 10 | 18.5 | 8.5 |
| *Neisseria* | -0.92787 | 0.228394 | 2 | 9 | 7 |
| *Ruminococcus* | 1.863327 | -0.53301 | 16 | 1 | 15 |
| *Streptococcus* | -1.10028 | 0.228394 | 1 | 9 | 8 |
| *Thermus* | -0.23664 | 0.229104 | 11 | 15.5 | 4.5 |
| *Weissella* | -0.01035 | 0.218223 | 13 | 3 | 10 |
|  |  |  |  |  |  |
| par_scheperjans | |  |  |  |  |
| Genera | Die_BW | Die_MB | Die_BWranked | Die_MBranked | distances |
| *Butyrivibrio* | 1.756585 | 0.547373 | 13 | 8 | 5 |
| *Enterobacter* | -0.65716 | 0.555165 | 5 | 9 | 4 |
| *Enterococcus* | -0.62371 | 0.592176 | 7 | 12.5 | 5.5 |
| *Finegoldia* | -0.39797 | 0.590228 | 10 | 11 | 1 |
| *Fusobacterium* | -0.39851 | 0.535686 | 9 | 7 | 2 |
| *Klebsiella* | -0.71296 | 0.414913 | 2 | 5 | 3 |
| *Lactobacillus* | -0.65729 | -1.41031 | 4 | 2 | 2 |
| *Lactococcus* | -0.66641 | 0.520102 | 3 | 6 | 3 |
| *Pluralibacter* | 1.142324 | -1.27201 | 12 | 3 | 9 |
| *Proteus* | -0.42519 | -3.45956 | 8 | 1 | 7 |
| *Salmonella* | -1.0585 | 0.21817 | 1 | 4 | 3 |
| *Serratia* | -0.64378 | 0.592176 | 6 | 12.5 | 6.5 |
| *Weissella* | -0.18951 | 0.580489 | 11 | 10 | 1 |
|  |  |  |  |  |  |
| nash_chan | |  |  |  |  |
| Genera | Die_BW | Die_MB | Die_BWranked | Die_MBranked | distances |
| *Bifidobacterium* | -0.2435 | 0.546836 | 6 | 2 | 4 |
| *Desulfovibrio* | -0.25486 | 0.619691 | 5 | 6.5 | 1.5 |
| *Enterococcus* | -0.48143 | 0.619691 | 4 | 6.5 | 2.5 |
| *Fusobacterium* | -0.21313 | -2.1589 | 7 | 1 | 6 |
| *Haemophilus* | -0.88599 | 0.587231 | 2 | 3 | 1 |
| *Kluyvera* | 1.368177 | 0.619691 | 8 | 6.5 | 1.5 |
| *Salmonella* | -1.12121 | 0.617527 | 1 | 4 | 3 |
| *Streptococcus* | -0.76506 | 0.619691 | 3 | 6.5 | 3.5 |
|  |  |  |  |  |  |
| nash_ob_baker | |  |  |  |  |
| Genera | Die_BW | Die_MB | Die_BWranked | Die_MBranked | distances |
| *Acidovorax* | -0.44551 | 0.884555 | 20 | 32 | 12 |
| *Acinetobacter* | -0.4854 | 0.641073 | 14 | 21 | 7 |
| *Actinomyces* | 1.891311 | 0.883731 | 32 | 28 | 4 |
| *Akkermansia* | -0.34527 | 0.882093 | 26 | 25 | 1 |
| *Arcobacter* | -0.43431 | -0.57748 | 24 | 7 | 17 |
| *Bifidobacterium* | -0.53856 | -0.01468 | 11 | 10 | 1 |
| *Campylobacter* | -0.45876 | 0.857058 | 18 | 23 | 5 |
| *Citrobacter* | -0.5597 | 0.883986 | 10 | 31 | 21 |
| *Clostridium* | -0.79623 | 0.093983 | 1 | 12 | 11 |
| *Corynebacterium* | -0.63776 | 0.584309 | 7 | 19 | 12 |
| *Desulfomicrobium* | 1.891299 | -3.22772 | 31 | 1 | 30 |
| *Desulfovibrio* | -0.62944 | 0.88329 | 8 | 26 | 18 |
| *Enterobacter* | -0.46997 | 0.88371 | 15 | 27 | 12 |
| *Enterococcus* | -0.6262 | 0.156086 | 9 | 13 | 4 |
| *Eubacterium* | -0.17396 | -0.9468 | 27 | 4 | 23 |
| *Finegoldia* | -0.43498 | -0.81966 | 23 | 6 | 17 |
| *Fusobacterium* | -0.52085 | -0.96818 | 12 | 3 | 9 |
| *Gardnerella* | 1.719385 | 0.15799 | 29 | 14 | 15 |
| *Haemophilus* | -0.52036 | 0.883853 | 13 | 29 | 16 |
| *Lactobacillus* | -0.66814 | 0.377517 | 5 | 17 | 12 |
| *Lactococcus* | -0.66193 | 0.88388 | 6 | 30 | 24 |
| *Leuconostoc* | -0.44859 | 0.368062 | 19 | 16 | 3 |
| *Mycoplasma* | -0.46735 | 0.343793 | 16 | 15 | 1 |
| *Parabacteroides* | -0.43502 | 0.863041 | 22 | 24 | 2 |
| *Porphyromonas* | -0.43951 | 0.051725 | 21 | 11 | 10 |
| *Pseudomonas* | -0.7401 | -0.85194 | 2 | 5 | 3 |
| *Ruminococcus* | 1.461203 | 0.617102 | 28 | 20 | 8 |
| *Salmonella* | -0.72645 | -0.33563 | 3 | 8 | 5 |
| *Selenomonas* | 1.891212 | 0.642482 | 30 | 22 | 8 |
| *Serratia* | -0.46161 | -2.98546 | 17 | 2 | 15 |
| *Staphylococcus* | -0.70009 | -0.22197 | 4 | 9 | 5 |
| *Weissella* | -0.34531 | 0.399053 | 25 | 18 | 7 |
|  |  |  |  |  |  |
| ParkinsonsMetaG | |  |  |  |  |
| Genera | Die_BW | Die_MB | Die_BWranked | Die_MBranked | distances |
| *Alcaligenes_faecalis* | 0.867015 | 0.150382 | 22 | 12.5 | 9.5 |
| *Bacteroides_fragilis* | 0.861199 | -0.32365 | 19 | 3 | 16 |
| *Brevibacterium_linens* | 0.663448 | 0.623672 | 16 | 21 | 5 |
| *Clostridium_perfringens* | 0.424984 | 0.148811 | 11 | 10 | 1 |
| *Enterobacter_cloacae* | -0.38928 | 0.150382 | 4 | 12.5 | 8.5 |
| *Lactobacillus_acidophilus* | 0.867015 | 0.307553 | 22 | 15 | 7 |
| *Lactobacillus_curvatus* | 0.867015 | 0.616991 | 22 | 20 | 2 |
| *Lactobacillus_rhamnosus* | 0.867015 | -0.02988 | 22 | 5 | 17 |
| *Lactobacillus_sakei* | 0.256314 | -0.51193 | 9.5 | 1 | 8.5 |
| *Leuconostoc_carnosum* | 0.663448 | 0.307742 | 16 | 16 | 0 |
| *Leuconostoc_gelidum* | 0.657632 | 0.466474 | 14 | 19 | 5 |
| *Pediococcus_acidilactici* | 0.459881 | 0.092139 | 13 | 9 | 4 |
| *Proteus_mirabilis* | -0.63357 | 0.150362 | 3 | 11 | 8 |
| *Pseudomonas_fluorescens* | 0.4308 | 0.466199 | 12 | 18 | 6 |
| *Pseudomonas_oleovorans* | 0.046931 | 0.308467 | 7 | 17 | 10 |
| *Serratia_liquefaciens* | 0.867015 | 0.62411 | 22 | 22 | 0 |
| *Staphylococcus_aureus* | -1.7212 | 0.624482 | 1 | 23 | 22 |
| *Staphylococcus_epidermidis* | 0.76814 | -0.17265 | 18 | 4 | 14 |
| *Streptococcus_anginosus* | 0.052747 | 0.303799 | 8 | 14 | 6 |
| *Streptococcus_gordonii* | -0.35439 | -0.48246 | 5 | 2 | 3 |
| *Streptococcus_oralis* | 0.663448 | -0.00799 | 16 | 6 | 10 |
| *Streptococcus_sanguinis* | -0.79642 | -0.00762 | 2 | 7 | 5 |
| *Streptococcus_thermophilus* | -0.15082 | 0.067077 | 6 | 8 | 2 |
| *Weissella_hellenica* | 0.256314 | 0.624522 | 9.5 | 24 | 14.5 |
| *Alcaligenes_faecalis* | 0.867015 | 0.150382 | 22 | 12.5 | 9.5 |
| *Bacteroides_fragilis* | 0.861199 | -0.32365 | 19 | 3 | 16 |

**Table S4**. Table of Die score calculation for separated healthy state controls and diseased state samples across 12 datasets.

| hiv_dinh Healthy | |  |  |  |  |
| --- | --- | --- | --- | --- | --- |
| Genera | Die_BW | Die_MB | Die_BWranked | Die_MBranked | distances |
| *Bacteroides* | -0.76357 | -0.33442 | 2 | 1 | 1 |
| *Clostridium* | -0.89387 | 0.561551 | 1 | 3 | 2 |
| *Cronobacter* | -0.32889 | 0.581532 | 5 | 6 | 1 |
| *Fusobacterium* | -0.33037 | 0.578299 | 4 | 4 | 0 |
| *Lactococcus* | -0.65847 | 0.582015 | 3 | 8 | 5 |
| *Prevotella* | 1.77432 | 0.4073 | 7 | 2 | 5 |
| *Pseudobutyrivibrio* | 1.774584 | 0.581822 | 8 | 7 | 1 |
| *Ruminococcus* | 1.145443 | 0.580857 | 6 | 5 | 1 |
|  |  |  |  |  |  |
| hiv_dinh Diseased | |  |  |  |  |
| Genera | Die_BW | Die_MB | Die_BWranked | Die_MBranked | distances |
| *Bifidobacterium* | -0.35079 | -0.25077 | 5 | 2 | 3 |
| *Clostridium* | -0.89387 | -0.88704 | 1 | 1 | 0 |
| *Cronobacter* | -0.32889 | 0.625641 | 8 | 7 | 1 |
| *Desulfovibrio* | -0.35876 | 0.62828 | 4 | 11 | 7 |
| *Eubacterium* | -0.32541 | 0.628252 | 9 | 10 | 1 |
| *Fusobacterium* | -0.33037 | 0.551666 | 6.5 | 5 | 1.5 |
| *Lactobacillus* | -0.80568 | 0.625441 | 2 | 6 | 4 |
| *Lactococcus* | -0.65847 | 0.628194 | 3 | 9 | 6 |
| *Pseudobutyrivibrio* | 1.774584 | 0.626846 | 11 | 8 | 3 |
| *Ruminococcus* | 1.145443 | 0.183278 | 10 | 3 | 7 |
| *Serratia* | -0.33037 | 0.189617 | 6.5 | 4 | 2.5 |
|  |  |  |  |  |  |
| asd_son Healthy | |  |  |  |  |
| Genera | Die_BW | Die_MB | Die_BWranked | Die_MBranked | distances |
| *Akkermansia* | -0.13825 | 0.474253 | 12 | 7 | 5 |
| *Campylobacter* | -0.30919 | -3.09099 | 6 | 1 | 5 |
| *Comamonas* | 2.788856 | -0.70967 | 13 | 3 | 10 |
| *Delftia* | -0.14879 | 0.495019 | 11 | 13 | 2 |
| *Desulfovibrio* | -0.29436 | -0.70635 | 8 | 4 | 4 |
| *Enterococcus* | -0.40166 | 0.494577 | 4 | 11 | 7 |
| *Haemophilus* | -0.52186 | 0.492368 | 3 | 9 | 6 |
| *Helicobacter* | -0.30599 | -0.75297 | 7 | 2 | 5 |
| *Lactobacillus* | -0.56456 | 0.488281 | 2 | 8 | 6 |
| *Parabacteroides* | -0.26136 | 0.091395 | 10 | 6 | 4 |
| *Prevotella* | 2.905835 | -0.68868 | 14 | 5 | 9 |
| *Ralstonia* | -0.39974 | 0.493915 | 5 | 10 | 5 |
| *Salmonella* | -0.75824 | 0.49513 | 1 | 14 | 13 |
| *Stenotrophomonas* | -0.27483 | 0.494798 | 9 | 12 | 3 |
|  |  |  |  |  |  |
| asd_son Diseased | |  |  |  |  |
| Genera | Die_BW | Die_MB | Die_BWranked | Die_MBranked | distances |
| *Acidovorax* | -0.17002 | 0.472108 | 16 | 16.5 | 0.5 |
| *Acinetobacter* | -0.34943 | -0.79026 | 9 | 3 | 6 |
| *Bifidobacterium* | -0.28478 | 0.409176 | 13 | 6 | 7 |
| *Campylobacter* | -0.30919 | 0.470363 | 11 | 10 | 1 |
| *Citrobacter* | -0.44306 | 0.47136 | 6 | 12 | 6 |
| *Clostridium* | -0.38986 | -0.78266 | 8 | 4 | 4 |
| *Comamonas* | 2.788856 | 0.472232 | 19 | 18.5 | 0.5 |
| *Eubacterium* | 0.328775 | -2.05269 | 17 | 1 | 16 |
| *Fusobacterium* | -0.26111 | 0.471734 | 15 | 13 | 2 |
| *Haemophilus* | -0.52186 | 0.467808 | 3 | 9 | 6 |
| *Helicobacter* | -0.30599 | 0.471983 | 12 | 15 | 3 |
| *Klebsiella* | -0.50046 | 0.472232 | 4 | 18.5 | 14.5 |
| *Lactobacillus* | -0.56456 | -0.79468 | 2 | 2 | 0 |
| *Lactococcus* | -0.45215 | 0.466438 | 5 | 8 | 3 |
| *Neisseria* | -0.33391 | 0.472108 | 10 | 16.5 | 6.5 |
| *Parabacteroides* | -0.26136 | 0.142933 | 14 | 5 | 9 |
| *Pluralibacter* | 2.203671 | 0.470675 | 18 | 11 | 7 |
| *Ralstonia* | -0.39974 | 0.471921 | 7 | 14 | 7 |
| *Streptococcus* | -0.7601 | 0.418834 | 1 | 7 | 6 |
|  |  |  |  |  |  |
| edd_singh Healthy | |  |  |  |  |
| Genera | Die_BW | Die_MB | Die_BWranked | Die_MBranked | distances |
| *Actinomyces* | 1.870618 | 0.544377 | 17 | 11 | 6 |
| *Bacteroides* | -0.56708 | -0.40515 | 8 | 1 | 7 |
| *Bifidobacterium* | -0.5167 | 0.538924 | 9 | 5 | 4 |
| *Clostridium* | -0.65441 | 0.46259 | 5 | 3 | 2 |
| *Desulfovibrio* | -0.57185 | 0.543769 | 7 | 7 | 0 |
| *Finegoldia* | -0.50899 | 0.544574 | 12 | 16 | 4 |
| *Haemophilus* | -0.77875 | 0.543812 | 4 | 8 | 4 |
| *Kluyvera* | 1.183656 | 0.544565 | 15 | 14 | 1 |
| *Leuconostoc* | -0.51392 | 0.544574 | 10 | 16 | 6 |
| *Parabacteroides* | -0.45619 | 0.403223 | 13 | 2 | 11 |
| *Porphyromonas* | -0.51091 | 0.544539 | 11 | 12.5 | 1.5 |
| *Proteus* | -0.57283 | 0.544539 | 6 | 12.5 | 6.5 |
| *Pseudomonas* | -0.90785 | 0.543041 | 1 | 6 | 5 |
| *Raoultella* | 1.183479 | 0.544574 | 14 | 16 | 2 |
| *Ruminococcus* | 1.606337 | 0.53502 | 16 | 4 | 12 |
| *Salmonella* | -0.87537 | 0.544077 | 2 | 9 | 7 |
| *Yersinia* | -0.84979 | 0.544351 | 3 | 10 | 7 |
|  |  |  |  |  |  |
| edd_singh Diseased | |  |  |  |  |
| Genera | Die_BW | Die_MB | Die_BWranked | Die_MBranked | distances |
| *Acidovorax* | -0.41157 | 0.535284 | 16 | 16 | 0 |
| *Actinobacillus* | -0.75167 | 0.526364 | 3 | 13 | 10 |
| *Aeromonas* | -0.68573 | 0.381106 | 4 | 5 | 1 |
| *Alcaligenes* | 1.764953 | 0.537311 | 21 | 19 | 2 |
| *Anaeroplasma* | 1.870618 | 0.538238 | 22.5 | 23 | 0.5 |
| *Bifidobacterium* | -0.5167 | 0.483331 | 11 | 9 | 2 |
| *Brucella* | -0.53191 | 0.538006 | 10 | 21 | 11 |
| *Corynebacterium* | -0.6289 | -2.08777 | 8 | 1 | 7 |
| *Dickeya* | 1.288923 | 0.53818 | 17 | 22 | 5 |
| *Leptotrichia* | 1.71199 | -0.7794 | 20 | 2 | 18 |
| *Leuconostoc* | -0.51392 | -0.77546 | 12 | 4 | 8 |
| *Lysinibacillus* | -0.51107 | 0.537137 | 14 | 18 | 4 |
| *Neisseria* | -0.63721 | 0.532098 | 7 | 15 | 8 |
| *Ochrobactrum* | -0.51246 | 0.528507 | 13 | 14 | 1 |
| *Pasteurella* | -0.67785 | 0.536963 | 6 | 17 | 11 |
| *Proteus* | -0.57283 | 0.512927 | 9 | 11 | 2 |
| *Providencia* | 1.289276 | 0.444005 | 18 | 6 | 12 |
| *Ruminococcus* | 1.606337 | 0.49422 | 19 | 10 | 9 |
| *Shewanella* | -0.83868 | 0.452924 | 2 | 7 | 5 |
| *Staphylococcus* | -0.67868 | 0.523179 | 5 | 12 | 7 |
| *Tannerella* | 1.870618 | 0.53789 | 22.5 | 20 | 2.5 |
| *Treponema* | -0.50961 | -0.77604 | 15 | 3 | 12 |
| *Yersinia* | -0.84979 | 0.464913 | 1 | 8 | 7 |
|  |  |  |  |  |  |
| autism_kb Healthy | |  |  |  |  |
| Genera | Die_BW | Die_MB | Die_BWranked | Die_MBranked | distances |
| *Akkermansia* | 0.123079 | 0.553651 | 10 | 6 | 4 |
| *Bacteroides* | -0.6447 | -2.89367 | 1 | 1 | 0 |
| *Bifidobacterium* | -0.28305 | -0.43388 | 7 | 5 | 2 |
| *Campylobacter* | -0.28448 | 0.610158 | 6 | 11 | 5 |
| *Citrobacter* | -0.26728 | 0.609773 | 9 | 10 | 1 |
| *Clostridium* | -0.41281 | 0.607467 | 3 | 9 | 6 |
| *Desulfovibrio* | -0.29141 | -0.52114 | 5 | 3 | 2 |
| *Enterobacter* | -0.26848 | 0.602854 | 8 | 8 | 0 |
| *Enterococcus* | -0.47979 | 0.600548 | 2 | 7 | 5 |
| *Eubacterium* | 0.306588 | -0.51576 | 11 | 4 | 7 |
| *Haemophilus* | -0.34252 | -1.62399 | 4 | 2 | 2 |
|  |  |  |  |  |  |
| autism_kb Diseased | |  |  |  |  |
| Genera | Die_BW | Die_MB | Die_BWranked | Die_MBranked | distances |
| *Akkermansia* | 0.123079 | -0.02261 | 13 | 9 | 4 |
| *Bacteroides* | -0.6447 | 0.579724 | 2 | 10 | 8 |
| *Bifidobacterium* | -0.28305 | -0.02953 | 7 | 4 | 3 |
| *Clostridium* | -0.41281 | -3.03532 | 4 | 2 | 2 |
| *Desulfovibrio* | -0.29141 | 0.620919 | 6 | 13.5 | 7.5 |
| *Eubacterium* | 0.306588 | -0.53738 | 14 | 3 | 11 |
| *Finegoldia* | -0.25665 | 0.620919 | 10 | 13.5 | 3.5 |
| *Fusobacterium* | -0.25712 | -0.02629 | 9 | 8 | 1 |
| *Haemophilus* | -0.34252 | -0.02813 | 5 | 5 | 0 |
| *Lactobacillus* | -0.706 | -3.77693 | 1 | 1 | 0 |
| *Lactococcus* | -0.55415 | 0.621007 | 3 | 15 | 12 |
| *Parabacteroides* | -0.25652 | 0.605142 | 11 | 11 | 0 |
| *Pasteurella* | -0.2779 | -0.02682 | 8 | 7 | 1 |
| *Prevotella* | 3.473963 | 0.620831 | 15 | 12 | 3 |
| *Weissella* | 0.12275 | -0.02804 | 12 | 6 | 6 |
|  |  |  |  |  |  |
| hiv_noguerajulian Healthy | | |  |  |  |
| Genera | Die_BW | Die_MB | Die_BWranked | Die_MBranked | distances |
| *Bifidobacterium* | -0.70223 | 0.446427 | 5 | 5 | 0 |
| *Clostridium* | -0.79921 | 0.102334 | 3 | 2 | 1 |
| *Elusimicrobium* | -0.50643 | 0.434701 | 11 | 4 | 7 |
| *Flavobacterium* | -0.64583 | 0.457428 | 7 | 11 | 4 |
| *Helicobacter* | -0.61943 | 0.456071 | 8 | 7 | 1 |
| *Leuconostoc* | -0.60753 | 0.457438 | 9 | 12.5 | 3.5 |
| *Parabacteroides* | -0.59981 | 0.389185 | 10 | 3 | 7 |
| *Rhizobium* | -0.71449 | 0.457438 | 4 | 12.5 | 8.5 |
| *Staphylococcus* | -0.88731 | 0.457376 | 2 | 10 | 8 |
| *Streptococcus* | -1.03044 | -0.70133 | 1 | 1 | 0 |
| *Tannerella* | 1.587266 | 0.457303 | 13 | 9 | 4 |
| *Treponema* | -0.6462 | 0.447768 | 6 | 6 | 0 |
| *Weissella* | -0.46101 | 0.457143 | 12 | 8 | 4 |
|  |  |  |  |  |  |
| hiv_noguerajulian Diseased | | |  |  |  |
| *Genera* | Die_BW | Die_MB | Die_BWranked | Die_MBranked | distances |
| *Achromobacter* | 1.541777 | 0.322562 | 15 | 13 | 2 |
| *Acinetobacter* | -0.6799 | 0.322629 | 5 | 14 | 9 |
| *Aggregatibacter* | -0.14318 | 0.322028 | 9 | 9 | 0 |
| *Anaerobiospirillum* | 1.587266 | 0.313945 | 16.5 | 6 | 10.5 |
| *Brevibacterium* | 1.405225 | 0.322896 | 14 | 16 | 2 |
| *Ensifer* | 1.587266 | 0.322228 | 16.5 | 11 | 5.5 |
| *Enterococcus* | -0.74683 | 0.256028 | 3 | 3 | 0 |
| *Finegoldia* | -0.59952 | 0.322362 | 6 | 12 | 6 |
| *Gardnerella* | 1.359788 | 0.322161 | 12 | 10 | 2 |
| *Leptotrichia* | 1.405087 | 0.322763 | 13 | 15 | 2 |
| *Porphyromonas* | -0.55744 | 0.31341 | 7 | 5 | 2 |
| *Raoultella* | 1.086502 | 0.321627 | 10 | 8 | 2 |
| *Rhizobium* | -0.71449 | 0.32303 | 4 | 17 | 13 |
| *Ruminococcus* | 1.314122 | -4.94694 | 11 | 1 | 10 |
| *Salmonella* | -0.97666 | 0.320157 | 2 | 7 | 5 |
| *Streptococcus* | -1.03044 | -0.80718 | 1 | 2 | 1 |
| *Weissella* | -0.46101 | 0.274732 | 8 | 4 | 4 |
|  |  |  |  |  |  |
| cdi_schubert Healthy | | |  |  |  |
| Genera | Die_BW | Die_MB | Die_BWranked | Die_MBranked | distances |
| *Actinomyces* | 1.799697 | 0.349855 | 8.5 | 5 | 3.5 |
| *Anaerobiospirillum* | 1.799697 | 0.411455 | 8.5 | 7 | 1.5 |
| *Bradyrhizobium* | -0.67857 | 0.421721 | 4 | 8 | 4 |
| *Comamonas* | 1.799672 | 0.236922 | 7 | 3.5 | 3.5 |
| *Corynebacterium* | -0.73216 | -0.91294 | 2 | 1 | 1 |
| *Kluyvera* | 1.250672 | 0.041856 | 6 | 2 | 4 |
| *Neisseria* | -0.73895 | 0.452521 | 1 | 9 | 8 |
| *Proteus* | -0.72742 | 0.236922 | 3 | 3.5 | 0.5 |
| *Xanthomonas* | -0.55481 | 0.360121 | 5 | 6 | 1 |
|  |  |  |  |  |  |
| cdi_schubert Diseased | | |  |  |  |
| Genera | Die_BW | Die_MB | Die_BWranked | Die_MBranked | distances |
| *Acinetobacter* | -0.65072 | 0.235374 | 4 | 10 | 6 |
| *Campylobacter* | -0.59294 | 0.219358 | 5 | 4 | 1 |
| *Carnobacterium* | 1.799697 | 0.226638 | 11 | 6 | 5 |
| *Edwardsiella* | -0.77081 | 0.235374 | 3 | 10 | 7 |
| *Eikenella* | 0.335668 | 0.235374 | 9 | 10 | 1 |
| *Enterococcus* | -0.5882 | -5.30895 | 6 | 1 | 5 |
| *Finegoldia* | -0.4897 | 0.219904 | 7 | 5 | 2 |
| *Providencia* | 1.387859 | 0.233554 | 10 | 7 | 3 |
| *Pseudomonas* | -1.02073 | 0.169671 | 1 | 2 | 1 |
| *Shewanella* | -0.95889 | 0.234828 | 2 | 8 | 6 |
| *Weissella* | -0.21386 | 0.211896 | 8 | 3 | 5 |
|  |  |  |  |  |  |
| ob_zupancic Healthy | | |  |  |  |
| Genera | Die_BW | Die_MB | Die_BWranked | Die_MBranked | distances |
| *Acidovorax* | -0.47356 | 0.34387 | 9 | 14 | 5 |
| *Citrobacter* | -0.76085 | 0.341452 | 4 | 7 | 3 |
| *Dickeya* | 1.633865 | 0.343601 | 15 | 8 | 7 |
| *Enterobacter* | -0.76193 | 0.34372 | 3 | 9.5 | 6.5 |
| *Enterococcus* | -0.76852 | 0.34378 | 2 | 11 | 9 |
| *Eubacterium* | 0.21848 | -1.20663 | 13 | 3 | 10 |
| *Finegoldia* | -0.52847 | -1.20651 | 7 | 4 | 3 |
| *Leuconostoc* | -0.54002 | 0.34387 | 6 | 14 | 8 |
| *Pasteurella* | -0.68791 | -2.76131 | 5 | 1 | 4 |
| *Porphyromonas* | -0.38382 | 0.34387 | 12 | 14 | 2 |
| *Pseudomonas* | -0.99859 | 0.032605 | 1 | 5 | 4 |
| *Raoultella* | 1.410627 | -1.20717 | 14 | 2 | 12 |
| *Rhodococcus* | -0.38848 | 0.34372 | 11 | 9.5 | 1.5 |
| *Sphingomonas* | -0.52803 | 0.34384 | 8 | 12 | 4 |
| *Treponema* | -0.45546 | 0.332109 | 10 | 6 | 4 |
|  |  |  |  |  |  |
| ob_zupancic Diseased | | |  |  |  |
| Genera | Die_BW | Die_MB | Die_BWranked | Die_MBranked | distances |
| *Aggregatibacter* | 0.144164 | 0.33414 | 11 | 10 | 1 |
| *Akkermansia* | -0.30203 | 0.326515 | 9 | 7 | 2 |
| *Bacteroides* | -0.61421 | -5.79665 | 4 | 1 | 3 |
| *Bifidobacterium* | -0.54471 | -1.71701 | 5 | 3 | 2 |
| *Brachyspira* | -0.00457 | 0.334404 | 10 | 14.5 | 4.5 |
| *Citrobacter* | -0.76085 | 0.333655 | 2 | 8 | 6 |
| *Delftia* | -0.45826 | 0.33436 | 7 | 12 | 5 |
| *Eubacterium* | 0.21848 | 0.334404 | 12 | 14.5 | 2.5 |
| *Haemophilus* | -0.80736 | 0.333919 | 1 | 9 | 8 |
| *Lactobacillus* | -0.72971 | 0.322107 | 3 | 6 | 3 |
| *Leuconostoc* | -0.54002 | 0.334448 | 6 | 16 | 10 |
| *Parabacteroides* | -0.45424 | 0.181858 | 8 | 5 | 3 |
| *Pseudobutyrivibrio* | 1.931969 | 0.33436 | 16 | 12 | 4 |
| *Ruminococcus* | 1.559598 | 0.014018 | 14 | 4 | 10 |
| *Selenomonas* | 1.931883 | -1.79172 | 15 | 2 | 13 |
| *Sphingobium* | 1.411084 | 0.33436 | 13 | 12 | 1 |
|  |  |  |  |  |  |
| mhe_zhang Healthy | |  |  |  |  |
| Genera | Die_BW | Die_MB | Die_BWranked | Die_MBranked | distances |
| *Akkermansia* | -0.01016 | 0.330999 | 3 | 4 | 1 |
| *Citrobacter* | -0.68184 | 0.13331 | 2 | 2 | 0 |
| *Dickeya* | 1.863475 | 0.351809 | 6 | 5.5 | 0.5 |
| *Enterobacter* | -0.79275 | 0.216548 | 1 | 3 | 2 |
| *Herbaspirillum* | 1.53308 | 0.351809 | 4 | 5.5 | 1.5 |
| *Ruminococcus* | 1.863327 | -3.68521 | 5 | 1 | 4 |
|  |  |  |  |  |  |
| mhe_zhang Diseased | |  |  |  |  |
| Genera | Die_BW | Die_MB | Die_BWranked | Die_MBranked | distances |
| *Aggregatibacter* | 0.319645 | 0.002521 | 7 | 2.5 | 4.5 |
| *Citrobacter* | -0.68184 | 0.219455 | 5 | 11 | 6 |
| *Dickeya* | 1.863476 | 0.219298 | 11 | 9 | 2 |
| *Eikenella* | 0.872093 | 0.219455 | 8 | 11 | 3 |
| *Haemophilus* | -0.83397 | 0.21569 | 4 | 5 | 1 |
| *Herbaspirillum* | 1.53308 | 0.219455 | 9 | 11 | 2 |
| *Lactococcus* | -0.8526 | 0.218827 | 3 | 8 | 5 |
| *Leptotrichia* | 2.083517 | 0.218357 | 12 | 7 | 5 |
| *Leuconostoc* | -0.47035 | 0.002521 | 6 | 2.5 | 3.5 |
| *Neisseria* | -0.92787 | 0.217572 | 2 | 6 | 4 |
| *Ruminococcus* | 1.863327 | 0.186044 | 10 | 4 | 6 |
| *Streptococcus* | -1.10028 | -4.99181 | 1 | 1 | 0 |
|  |  |  |  |  |  |
| par_scheperjans Healthy | | |  |  |  |
| Genera | Die_BW | Die_MB | Die_BWranked | Die_MBranked | distances |
| *Akkermansia* | -0.18934 | 0.623408 | 13 | 10 | 3 |
| *Bifidobacterium* | -0.41577 | 0.623443 | 8 | 11.5 | 3.5 |
| *Comamonas* | 1.961075 | 0.623355 | 15 | 9 | 6 |
| *Enterobacter* | -0.65716 | 0.623285 | 6 | 8 | 2 |
| *Finegoldia* | -0.39797 | 0.623567 | 11 | 14 | 3 |
| *Haemophilus* | -0.72069 | 0.620886 | 3 | 6 | 3 |
| *Hafnia* | 1.961195 | -0.77588 | 16 | 4 | 12 |
| *Klebsiella* | -0.71296 | 0.622808 | 4 | 7 | 3 |
| *Lactococcus* | -0.66641 | -0.77673 | 5 | 3 | 2 |
| *Leuconostoc* | -0.41318 | 0.623585 | 9 | 15.5 | 6.5 |
| *Pluralibacter* | 1.142324 | -2.18509 | 14 | 1 | 13 |
| *Porphyromonas* | -0.40434 | 0.623443 | 10 | 11.5 | 1.5 |
| *Salmonella* | -1.0585 | -0.7856 | 1 | 2 | 1 |
| *Serratia* | -0.64378 | 0.623585 | 7 | 15.5 | 8.5 |
| *Streptococcus* | -0.90472 | 0.61279 | 2 | 5 | 3 |
| *Weissella* | -0.18951 | 0.623479 | 12 | 13 | 1 |
|  |  |  |  |  |  |
| par_scheperjans Diseased | | |  |  |  |
| Genera | Die_BW | Die_MB | Die_BWranked | Die_MBranked | distances |
| *Acidovorax* | -0.30755 | 0.700444 | 13 | 12 | 1 |
| *Akkermansia* | -0.18934 | 0.698075 | 14 | 11 | 3 |
| *Bifidobacterium* | -0.41577 | -1.30696 | 10 | 2 | 8 |
| *Butyrivibrio* | 1.756585 | 0.700862 | 17 | 13 | 4 |
| *Campylobacter* | -0.42053 | -0.29908 | 9 | 5 | 4 |
| *Citrobacter* | -0.66238 | 0.70351 | 4 | 14 | 10 |
| *Clostridium* | -0.60416 | -0.2765 | 7 | 7 | 0 |
| *Enterobacter* | -0.65716 | 0.704067 | 5 | 18 | 13 |
| *Enterococcus* | -0.62371 | -0.29768 | 6 | 6 | 0 |
| *Hafnia* | 1.961195 | 0.703928 | 19 | 15.5 | 3.5 |
| *Klebsiella* | -0.71296 | -0.31106 | 3 | 4 | 1 |
| *Leuconostoc* | -0.41318 | 0.704067 | 11 | 18 | 7 |
| *Parabacteroides* | -0.39913 | 0.240682 | 12 | 8 | 4 |
| *Pluralibacter* | 1.142324 | 0.630762 | 16 | 10 | 6 |
| *Prevotella* | 1.961075 | -3.53873 | 18 | 1 | 17 |
| *Proteus* | -0.42519 | -1.29943 | 8 | 3 | 5 |
| *Pseudobutyrivibrio* | 1.961204 | 0.704067 | 20 | 18 | 2 |
| *Pseudomonas* | -0.857 | 0.704207 | 2 | 20 | 18 |
| *Raoultella* | 1.141626 | 0.703928 | 15 | 15.5 | 0.5 |
| *Streptococcus* | -0.90472 | 0.57195 | 1 | 9 | 8 |
|  |  |  |  |  |  |
| nash_chan Healthy | |  |  |  |  |
| Genera | Die_BW | Die_MB | Die_BWranked | Die_MBranked | distances |
| *Akkermansia* | -0.20256 | 0.428528 | 5 | 6 | 1 |
| *Bifidobacterium* | -0.2435 | 0.43934 | 3 | 7 | 4 |
| *Clostridium* | -0.39519 | 0.33637 | 2 | 5 | 3 |
| *Haemophilus* | -0.88599 | -0.80094 | 1 | 1 | 0 |
| *Kluyvera* | 1.368177 | -0.7546 | 6 | 2 | 4 |
| *Parabacteroides* | -0.21348 | -0.16973 | 4 | 4 | 0 |
| *Prevotella* | 2.040372 | -0.72989 | 8 | 3 | 5 |
| *Ruminococcus* | 1.59174 | 0.468687 | 7 | 8 | 1 |
|  |  |  |  |  |  |
| nash_chan Diseased | |  |  |  |  |
| Genera | Die_BW | Die_MB | Die_BWranked | Die_MBranked | distances |
| *Bifidobacterium* | -0.2435 | 0.425125 | 6 | 2 | 4 |
| *Desulfovibrio* | -0.25486 | 0.439926 | 5 | 5 | 0 |
| *Enterococcus* | -0.48143 | 0.439926 | 4 | 5 | 1 |
| *Klebsiella* | -0.90587 | 0.360988 | 2 | 1 | 1 |
| *Salmonella* | -1.12121 | 0.436226 | 1 | 3 | 2 |
| *Streptococcus* | -0.76506 | 0.439926 | 3 | 5 | 2 |
|  |  |  |  |  |  |
| nash_ob_baker Healthy | | |  |  |  |
| Genera | Die_BW | Die_MB | Die_BWranked | Die_MBranked | distances |
| *Bifidobacterium* | -0.53856 | -1.09881 | 8 | 3 | 5 |
| *Citrobacter* | -0.5597 | 0.812008 | 7 | 12 | 5 |
| *Clostridium* | -0.79623 | -2.7805 | 2 | 1 | 1 |
| *Desulfovibrio* | -0.62944 | -1.09166 | 6 | 4 | 2 |
| *Haemophilus* | -0.52036 | 0.810692 | 9 | 10 | 1 |
| *Lactococcus* | -0.66193 | -0.45233 | 5 | 6 | 1 |
| *Parabacteroides* | -0.43502 | 0.095992 | 10 | 7 | 3 |
| *Prevotella* | 1.891218 | 0.69497 | 13 | 9 | 4 |
| *Pseudomonas* | -0.7401 | 0.812337 | 3 | 13 | 10 |
| *Ruminococcus* | 1.461203 | -1.2959 | 12 | 2 | 10 |
| *Staphylococcus* | -0.70009 | 0.18109 | 4 | 8 | 4 |
| *Streptococcus* | -0.94065 | -0.58125 | 1 | 5 | 4 |
| *Weissella* | -0.34531 | 0.81192 | 11 | 11 | 0 |
|  |  |  |  |  |  |
| nash_ob_baker Diseased | | |  |  |  |
| Genera | Die_BW | Die_MB | Die_BWranked | Die_MBranked | distances |
| *Acidovorax* | -0.44551 | -0.21057 | 20 | 8 | 12 |
| *Acinetobacter* | -0.4854 | 0.676767 | 15 | 19 | 4 |
| *Actinomyces* | 1.891311 | 0.674465 | 31 | 18 | 13 |
| *Arcobacter* | -0.43431 | 1.106371 | 24 | 30 | 6 |
| *Bacteroides* | -0.61972 | 1.104707 | 10 | 25 | 15 |
| *Bifidobacterium* | -0.53856 | -0.67308 | 12 | 5 | 7 |
| *Brevibacterium* | 1.633316 | -0.18752 | 27 | 11 | 16 |
| *Campylobacter* | -0.45876 | 1.057085 | 18 | 24 | 6 |
| *Citrobacter* | -0.5597 | 0.677397 | 11 | 20 | 9 |
| *Clostridium* | -0.79623 | 0.216656 | 2 | 15 | 13 |
| *Comamonas* | 1.891218 | 1.106038 | 30 | 28 | 2 |
| *Corynebacterium* | -0.63776 | -1.12189 | 7 | 4 | 3 |
| *Desulfovibrio* | -0.62944 | 1.105495 | 8 | 27 | 19 |
| *Enterobacter* | -0.46997 | 1.105075 | 16 | 26 | 10 |
| *Enterococcus* | -0.6262 | 0.678588 | 9 | 21 | 12 |
| *Eubacterium* | -0.17396 | -0.20036 | 26 | 9 | 17 |
| *Finegoldia* | -0.43498 | -0.22011 | 23 | 7 | 16 |
| *Fusobacterium* | -0.52085 | 0.154292 | 13 | 14 | 1 |
| *Gardnerella* | 1.719385 | 0.678693 | 28 | 22 | 6 |
| *Haemophilus* | -0.52036 | -1.17775 | 14 | 3 | 11 |
| *Lactobacillus* | -0.66814 | -0.19672 | 5 | 10 | 5 |
| *Lactococcus* | -0.66193 | -0.17915 | 6 | 12 | 6 |
| *Leuconostoc* | -0.44859 | -0.60766 | 19 | 6 | 13 |
| *Mycoplasma* | -0.46735 | 0.678921 | 17 | 23 | 6 |
| *Parabacteroides* | -0.43502 | 0.246148 | 22 | 16 | 6 |
| *Porphyromonas* | -0.43951 | -2.14493 | 21 | 1 | 20 |
| *Salmonella* | -0.72645 | 1.106379 | 3 | 31 | 28 |
| *Selenomonas* | 1.891212 | 1.106222 | 29 | 29 | 0 |
| *Staphylococcus* | -0.70009 | 0.118016 | 4 | 13 | 9 |
| *Streptococcus* | -0.94065 | 0.662393 | 1 | 17 | 16 |
| *Weissella* | -0.34531 | -1.50146 | 25 | 2 | 23 |
|  |  |  |  |  |  |
| ParkinsonsMetaG Control |  |  |  |  |  |
| Genera | Die_BW | Die_MB | Die_BWranked | Die_MBranked | distances |
| *Clostridium_perfringens* | 0.424984 | 0.021231 | 8 | 6 | 2 |
| *Enterobacter_cloacae* | -0.38928 | 1.000725 | 3 | 16 | 13 |
| *Lactobacillus_acidophilus* | 0.867015 | 1.000692 | 15 | 15 | 0 |
| *Lactobacillus_curvatus* | 0.867015 | 0.591137 | 15 | 10 | 5 |
| *Lactobacillus_rhamnosus* | 0.867015 | 0.604172 | 15 | 12 | 3 |
| *Lactobacillus_sakei* | 0.256314 | 0.320361 | 7 | 8 | 1 |
| *Leuconostoc_carnosum* | 0.663448 | 0.219138 | 11.5 | 7 | 4.5 |
| *Leuconostoc_gelidum* | 0.657632 | 0.411975 | 10 | 9 | 1 |
| *Pediococcus_acidilactici* | 0.459881 | 0.79881 | 9 | 14 | 5 |
| *Staphylococcus_aureus* | -1.7212 | 0.610851 | 1 | 13 | 12 |
| *Staphylococcus_epidermidis* | 0.76814 | -0.75366 | 13 | 4 | 9 |
| *Streptococcus_anginosus* | 0.052747 | 0.604155 | 6 | 11 | 5 |
| *Streptococcus_gordonii* | -0.35439 | -1.33851 | 4 | 1 | 3 |
| *Streptococcus_oralis* | 0.663448 | -1.14404 | 11.5 | 2 | 9.5 |
| *Streptococcus_sanguinis* | -0.79642 | -0.16887 | 2 | 5 | 3 |
| *Streptococcus_thermophilus* | -0.15082 | -0.84111 | 5 | 3 | 2 |
|  |  |  |  |  |  |
| ParkinsonsMetaG Disease |  |  |  |  |  |
| Genera | Die_BW | Die_MB | Die_BWranked | Die_MBranked | distances |
| *Alcaligenes_faecalis* | 0.867015 | 0.41261 | 18 | 12 | 6 |
| *Bacteroides_fragilis* | 0.861199 | 0.568593 | 15 | 16 | 1 |
| *Brevibacterium_linens* | 0.663448 | 0.722508 | 12.5 | 17 | 4.5 |
| *Clostridium_perfringens* | 0.424984 | 0.095779 | 9 | 5 | 4 |
| *Lactobacillus_acidophilus* | 0.867015 | 0.411132 | 18 | 11 | 7 |
| *Lactobacillus_curvatus* | 0.867015 | 0.563562 | 18 | 13 | 5 |
| *Lactobacillus_rhamnosus* | 0.867015 | 0.210206 | 18 | 8 | 10 |
| *Lactobacillus_sakei* | 0.256314 | -0.15746 | 8 | 3 | 5 |
| *Leuconostoc_gelidum* | 0.657632 | 0.568551 | 11 | 14 | 3 |
| *Pediococcus_acidilactici* | 0.459881 | -0.35748 | 10 | 2 | 8 |
| *Proteus_mirabilis* | -0.63357 | 0.256589 | 3 | 9 | 6 |
| *Pseudomonas_oleovorans* | 0.046931 | 0.724498 | 6 | 20 | 14 |
| *Serratia_liquefaciens* | 0.867015 | 0.723405 | 18 | 18 | 0 |
| *Staphylococcus_aureus* | -1.7212 | 0.724393 | 1 | 19 | 18 |
| *Staphylococcus_epidermidis* | 0.76814 | -0.38838 | 14 | 1 | 13 |
| *Streptococcus_anginosus* | 0.052747 | 0.40071 | 7 | 10 | 3 |
| *Streptococcus_gordonii* | -0.35439 | -0.05509 | 4 | 4 | 0 |
| *Streptococcus_oralis* | 0.663448 | 0.100198 | 12.5 | 6 | 6.5 |
| *Streptococcus_sanguinis* | -0.79642 | 0.568588 | 2 | 15 | 13 |
| *Streptococcus_thermophilus* | -0.15082 | 0.136944 | 5 | 7 | 2 |

**Table S5**. Selected genera found in disease networks and their association with specific diseases/disorders (red colour denotes genera that are most likely to die while green colour denotes the opposite).

| Genera | Disease/Disorder | Dataset | Description and supporting reference |
| --- | --- | --- | --- |
| *Clostridium* | HIV | HIV_Dinh | In HIV patients, CDI is the leading cause of bacterial diarrhea^1^ |
| *Comamonas*, *Streptococcus* and *Prevotella*, *Lactobacillus* | autism (ASD) | asd_son and asd_kb | Elevated in ASD patients^2^ |
| *Yersinia* | enteric diarrheal disease (EDD) | edd_sinh | Associated with enteric infections^3^ |
| *Streptococcus* | HIV | hiv_noguerajulian | Its colonization is affected by HIV^4^ |
| *Pseudomonas* | clostridium difficile (CDI) | cd_schubert | Correlated with CDI in hospital correlation studies for infectious diseases^5^ |
| *Pseudobutyrivibrio* | obesity | ob_zupancic | Induced and elevated upon high-fat diet^6^ |
| *Leptotrichia* | liver disease | mhe_zhang | Associated with multiple liver disease including: Liver abscess, liver fluke infection, mild liver dysfunction^7^ |
| *Streptococcus* | Parkinson’s | par_scheperjans/ParkinsonsMetaG | Used as fecal microbiota transplant in Parkinson’s Disease^8^ |
| *Salmonella* | non-alcoholic steatohepatitis (NASH) | nash_chang | Induces microbiota-driven gut vascular barrier (GVB) disruption, which is a prerequisite for non-alcoholic steatohepatitis development^9^ |
| *Actinomyces* | non-alcoholic steatohepatitis (NASH) | nash_ob_baker | Induces proteolytic fermentation. Patients with NASH have dysregulation of branched-chain amino acids (BCAA) metabolism. BCAAs are produced by proteolytic fermentation in the colon^10^ |

**References**

1. Imlay, H., Kaul, D. & Rao, K. Risk factors for Clostridium difficile infection in HIV-infected patients . *SAGE Open Med.* (2016) doi:10.1177/2050312116684295.

2. Lee, Y. *et al.* Rapid assessment of microbiota changes in individuals with autism spectrum disorder using bacteria-derived membrane vesicles in urine. *Exp. Neurobiol.* **26**, 307–317 (2017).

3. Saraka, D. *et al.* Yersinia enterocolitica, a Neglected Cause of Human Enteric Infections in Côte d’Ivoire. *PLoS Negl. Trop. Dis.* **11**, (2017).

4. Gill, C. J. *et al.* Impact of Human Immunodeficiency Virus Infection on Streptococcus pneumoniae Colonization and Seroepidemiology among Zambian Women . *J. Infect. Dis.* **197**, 1000–1005 (2008).

5. Ricciardi, R. *et al.* Predictors of Clostridium dificile colitis infections in hospitals. *Epidemiol. Infect.* **136**, 913–921 (2008).

6. Lin, H., An, Y., Hao, F., Wang, Y. & Tang, H. Correlations of Fecal Metabonomic and Microbiomic Changes Induced by High-fat Diet in the Pre-Obesity State. *Sci. Rep.* **6**, (2016).

7. Eribe, E. R. K. & Olsen, I. *Leptotrichia* species in human infections II. *J. Oral Microbiol.* **9**, 1368848 (2017).

8. Dutta, S. K. *et al.* Parkinson’s Disease: The Emerging Role of Gut Dysbiosis, Antibiotics, Probiotics, and Fecal Microbiota Transplantation. *J. Neurogastroenterol. Motil.* **25**, 363–376 (2019).

9. Mouries, J. *et al.* Microbiota-driven gut vascular barrier disruption is a prerequisite for non-alcoholic steatohepatitis development. *J. Hepatol.* (2019) doi:10.1016/j.jhep.2019.08.005.

10. Zhou, D. & Fan, J. G. Microbial metabolites in non-alcoholic fatty liver disease. *World J. Gastroenterol.* **25**, 2019–2028 (2019).
